# Mechanistic insight into copper cation exchange in cadmium selenide semiconductor nanocrystals using X-ray absorption spectroscopy

**DOI:** 10.1038/s41467-020-20712-0

**Published:** 2021-01-19

**Authors:** Alex Khammang, Joshua T. Wright, Robert W. Meulenberg

**Affiliations:** 1 Department of Physics and Astronomy and Frontier Institute for Research in Sensor Technologies, University of Maine, Orono, ME 04469 USA; 2 Department of Physics, Illinois Institute of Technology, Chicago, IL USA

**Keywords:** Materials chemistry, Quantum dots, Nanoscale materials

## Abstract

In terms of producing new advances in sustainable nanomaterials, cation exchange (CE) of post-processed colloidal nanocrystals (NCs) has opened new avenues towards producing non-toxic energy materials via simple chemical techniques. The main processes governing CE can be explained by considering hard/soft acid/base theory, but the detailed mechanism of CE, however, has been debated and has been attributed to both diffusion and vacancy processes. In this work, we have performed in situ x-ray absorption spectroscopy to further understand the mechanism of the CE of copper in solution phase CdSe NCs. The x-ray data indicates clear isosbestic points, suggestive of cooperative behavior as previously observed via optical spectroscopy. Examination of the extended x-ray absorption fine structure data points to the observation of interstitial impurities during the initial stages of CE, suggesting the diffusion process is the fundamental mechanism of CE in this system.

## Abstract (web summary)

There currently exists some debate as to exact mechanism of cation exchange in semiconductor nanocrystals, a crucial question regarding the fundamental materials chemistry of these systems. Here, the authors report a detailed investigation of the mechanism of cation exchange in nanocrystals, which unambiguously shows evidence of interstitial intermediates during the exchange process.

## Introduction

Since the first synthesis of high-quality nanocrystals (NCs) by hot-injection methods^1^, many research groups have sought to find comparable syntheses for a wide range of inorganic materials. Although a host of materials systems have been successfully synthesized^2–5^, the controllable synthesis of other systems, such as the Group IV NC system, has been difficult. The inability to make just about any binary NC system, however, was solved using the cation exchange (CE) approach. The first report of CE in NCs reported the fast transformation (<1 min) of CdSe NCs into Ag_2_Se by introducing a methanolic Au^+^ solution into a colloidal suspension of CdSe NCs^6^. The primary driving force (energetically) behind CE processes can be explained by considering hard/soft acid/base (HSAB) theory, although there are a host of parameters than can influence this process. Initially suggested by the Alivisatos group^6^, this model implies that the “hardness” or “softness” of the materials undergoing CE will drive the exchange process. For example, if we consider the well-known example of Cu^+^ cation exchange in CdSe NCs^7,8^ in the core NC we have a soft acid (cadmium cation, Cd^2+^) and a soft base (selenium anion, Se^2−^) with the incoming cation a soft acid (copper cation, Cu^+^). As the Cu^+^ ion is softer than the Cd^2+^ ion, with an appropriate hard base (such as methanol, MeOH), the CE process can be favored via a simple soft acid/soft base combination, Cu^+^/Se^2−^.

Since the early reports of complete structural conversion via CE, researchers have shown that partial exchange of the NC lattice is possible, but the models behind the partial exchange process are debated. One model (that we will call the interstitial model) suggests that partial cation exchange in a AB lattice (where A is a cation and B is an anion) is governed by a multi-step process that begins with the diffusion of the impurity ion (M^n+^) into NC with the impurity ion sitting on an interstitial site^9^. If more than one impurity ion is introduced to the NC, the strong Coulomb interaction between the two M^n+^ force the ions to the surface. Following surface segregation, the M^n+^ can substitute for a host cation via a kick-off reaction, where M^+^ substitutes for the host cation site (substitutionally) leading to an M^−^ site. This new negatively changed site can accumulate the interstitial M^n+^ ions leading to local pockets of “MB” material. This process can repeat until the formation of the M_2_B material. Another model (that we will call the vacancy model) for the CE process in NCs is a vacancy driven formation^10^. In this case, the NC must be imperfect, meaning it must possess surface vacancies. With the existing surface vacancy, a host cation transfers to the vacant surface site, and a surface M^n+^ ion hops to the vacancy left behind by the host cation. The host cation, now on the surface, desorbs as a metal complex and a “new” M^n+^ ion adsorbs onto the NC surface. This process continues to completion. In addition, it has been recently suggested that CE in CdSe (with Ag or Cu) exhibits positive cooperativity, meaning that only two phases are present during the CE process^7^.

While there have been a large number of studies that investigate the possible mechanism for CE in NCs, there still exists some debate on the exact mechanism as the process seems to be highly dependent on a number of factors including crystal type, particle shape, and surface ligand type^8–13^. For cation exchange of Cu in CdSe NCs, it has been generally suggested that the process may proceed via an interstitial mechanism, but there has been no direct evidence of this. In this work, we investigate the mechanism of cation exchange in CdSe NCs using in situ x-ray absorption spectroscopy (XAS). The use of *operando* or in situ XAS for probing catalytic materials has been well documented^14–17^, but progress in applying these techniques related to studying the complex chemistry and physics of colloidal nanostructures has been slower. One of the earliest uses of in situ XAS was by the Alivisatos group^6^ which showed that Ag^+^ cation exchange in CdSe NCs can be described as a rapid transformation between two species, CdSe and Ag_2_Se, with no contributions from impurity phases, yet no detailed mechanism for the process was ascertained. Kompch and co-workers have performed the most detailed XAS work on CE in CdSe^18^ where the mechanism of Ag CE in CdSe NC was probed via reverse Monte Carlo analysis of the extended x-ray absorption fine structure (EXAFS) data, although this study focused on solid-phase materials. Inspired by this work and the work of the Jain group^7^, in our current work, we use partial cation exchange of Cu to probe various stages of the CE reaction in solution phase CdSe NCs. Analysis of the EXAFS data show evidence for interstitials during the CE process. In addition, our XAS results suggest the CE process exhibits positive cooperativity, in agreement with prior results^7^. Our measurements are the first solution based measurements that provide structural evidence for interstitial inclusions and show that in situ XAS is a powerful and local probe to investigate chemical mechanisms in NCs.

## Results

### Copper cation exchange in CdSe NCs as probed by X-ray absorption spectroscopy

The CdSe NCs were synthesized through a colloidal hot-injection method as previously reported^19,20^ (see Methods for details). These synthetic methods can produce wurtzite CdSe NCs with highly tunable sizes; in this work, we have studied three distinct (nominal) sizes of NCs: 4.0, 5.0, and 6.0 nm diameter. We also attempted measurements on 3.0 nm CdSe NCs. Due to both air and beam instability, high-quality EXAFS data was not attainable. But x-ray absorption near edge structure (XANES) data was obtained and shows the same general trends as the other particle sizes (see Fig. S4). The cation exchange reaction is initiated by adding an appropriate molar fraction of the metal salt to the QD solution^7^. Because the reaction is reactant limited, a sub-stoichiometric amount of the metal cation salt (in this work we used tetrakis(acetonitrile)copper(I) hexafluorophosphate, Cu(I)PF_6_; see Methods section) can be added offering the ability to probe various stages of the chemical reaction. The exact details of the cation salt loading are shown in Supplementary Table S1. As the ethanolic copper salt solution is added to the NC solution, x-ray absorption fine structure (XAFS) measurements were conducted at the Cd, Se, and Cu K-edges. We also attempted some XAFS measurements of Ag cation exchange in CdSe. These results, while preliminary, are qualitatively similar to our observations for Cu cation exchange (Figures S12 and S13). It should be noted that the kinetics of this reaction (less than one second) are too fast to monitor via traditional XAFS setups without using microfluidic reactors^21^. Attempts to use metal salts that slowed the cation exchange reaction^22^ to allow kinetic XAFS studies were unsuccessful.

**Fig. 1:**
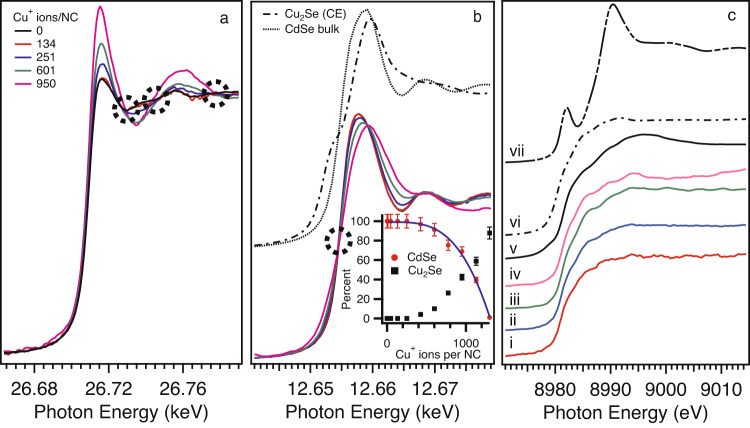
Core level XANES during the cation exchange reaction. X-ray absorption spectra for 5.0 nm CdSe NCs at the (**a**) Cd K-edge, (**b**) Se K-edge, and (**c**) Cu K-edge for (i) 134, (ii) 251, (iii) 601, (iv) 950 Cu ions/NC, (v) Cu_2_Se via cation exchange of CdSe, (vi) commercial Cu_2_Se, and (vii) Cu(I)PF_6_. The inset of (**b**) are the LCF results for the Se K-edge using a CdSe and Cu_2_Se model. The error bars in the inset represent uncertainties in the LCF.

In Fig. 1, representative XANES spectra are displayed for 5.0 nm CdSe NCs. XANES spectra for other sizes can be found in the Supplementary Information. At the lowest Cu salt loading levels (mol Cu/mol Cd = 0.01; ~12 Cu atoms/NC), no distinct changes in the Cd or Se K-edge XANES features are evident, and there is no detectable signal for the Cu K-edge. This is not totally unexpected, as the equivalent amount of Cu present in the interrogation volume of the XAFS experiment is ~2 nmol. At the second concentration step (mol Cu/mol Cd = 0.04; ~47 Cu atoms/NC), we observe a very weak Cu K-edge signal, with no strong changes in the Cd or Se K-edges (SI Figure S5). It is noteworthy that the Cu K-edge spectrum for ~47 Cu ions/NC is distinct from the spectrum for Cu(I)PF_6_ (Fig. 1c,vii) suggesting incorporation of Cu into the CdSe NC. As the amount of Cu in the CdSe solution is continually increased, subsequent changes in the XANES features are evident. Both the Cd and Se K-edges show gradual changes in the XANES with increasing copper concentration with clear existence of isosbestic points (dotted circles in Fig. 1a and b). As increasing amounts of Cu are added to the CdSe solution, transformation of the CdSe species to Cu_2_Se is evident via Se K-edge XANES (Fig. 1b). Principal component analysis of the Se K-edge XANES suggests only two species are present (consistent with the isosbestic points) and linear combination fitting (LCF) indicates complete transformation of the CdSe → Cu_2_Se for Cu mol% > 50%. Below a concentration of ~ 600 Cu ions/NC, there is a very small fraction of Cu_2_Se which is similar to observations by the Jain group who used optical spectroscopy to probe the CE reaction^7^. The CdSe fraction as a function of Cu concentration can be fit to the Hill equation (solid line in the inset of Fig. 1b) with a Hill coefficient of *n* = 3.5, which is consistent with positive cooperativity and the results from the Jain group. Meanwhile, with increasing Cu concentration the Cd K-edge XANES exhibits a transformation from CdSe to a five or six-fold coordinated Cd species (vide infra). This behavior is strongly suggestive of cooperative behavior consistent with the LCF and again supports conclusions by the Jain group^7^.

### Cation exchange inclusion models: Vacancy mediated versus interstitial inclusions

**Fig. 2:**
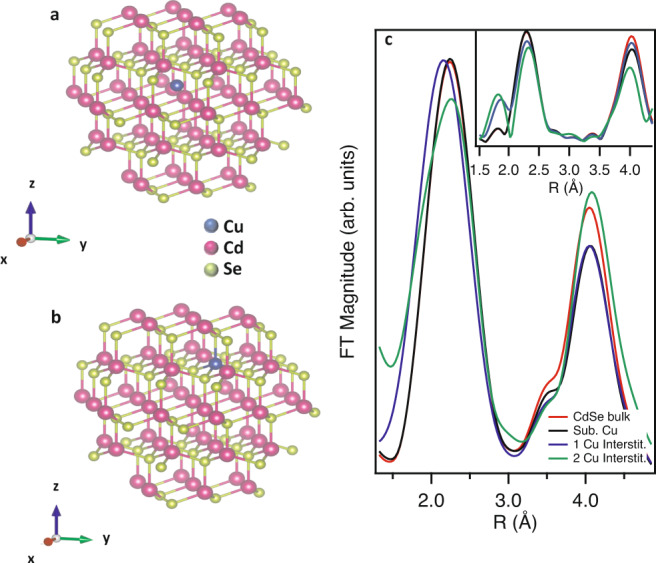
Structural models of Cu atom inclusion in CdSe and theoretical Fourier transformed EXAFS data. CdSe cluster model showing (**a**) substitutional and (**b**) interstitial Cu dopants and (**c**) theoretical Fourier transformed EXAFS magnitude for the Cd K-edge for CdSe bulk and substitutional and interstitial Cu dopants. See text for differences between (**c**) and the inset for (**c**).

When considering possible mechanisms for CE in NCs, the two most common candidates are diffusion and vacancy models. For low Cu concentrations (i.e., partial CE), we would expect a significant fraction of Cu atoms in interstitial lattice sites for a pure diffusion model, while pre-existing vacancies can allow for direct substitutional incorporation of the Cu dopant. To ascertain the plausibility of the two models, we constructed cluster models of CdSe (125 atoms) and consider four cases: (a) undoped CdSe, (b) a substitutional Cu dopant, (c) an interstitial Cu dopant, and (d) two interstitial Cu dopants. (Figure 2a). We then computed the theoretical EXAFS spectra for the Cd K-edge using FEFF9^23^. In the FEFF calculations, for self-consistency, we used a 5 Å cluster and considered the effects of a core hole using the final state rule. Figure 2c displays the Fourier transformed (FT) EXAFS magnitude for the various cluster models. We note that the FEFF calculation allows the extraction of EXAFS data out to *k* = 20 Å^−1^, and while good experimental EXAFS data can extend to *k* = 15 Å^−1^, in the case of NC samples (i.e., like we are studying) *k* = 10–12 Å^−1^ is typical. We therefore display the theoretical FT EXAFS data with a transform range 3 ≤ *k *≤ 15 Å^−1^ in the inset of Fig. 2c. For a substitutional Cu impurity, very little deviation from the undoped spectrum is observed. This is not surprising, as the main scattering peak is related to the nearest neighbors (NN), and the Cu substitutional impurity sits in a next-nearest neighbor (NNN) lattice position. This explains why there is a change to the scattering peak at ~4.0 Å as that peak is related to NNN contributions. Realistically, however, we cannot access these features for our NC samples, so we will focus solely on analysis of the main scattering peak (NN contributions). For interstitial impurities, of particular note is the appearance of scattering features at values of *R* ~ 1.9 Å (not phase shift corrected). Since a FT window of 3 ≤ *k *≤ 15 Å^−1^ is not experimentally feasible in our system, we also plot the theoretical FT EXAFS data for a FT window of 3 ≤ *k *≤ 10 Å^−1^ (Fig. 2c), which should allow a more direct comparison to our experimental data. Of note is the smearing of the low *R* scattering feature into the main peak which results in a single peak that is shifted slightly to lower *R* (by ~0.06 Å) with a broadening of the main peak (~10%) due to the appearance of interstitial Cu. As more Cu is introduced into the model, an increased broadening of the main peak is observed. We hypothesize, therefore, that visual inspection for broadening of the main scattering peak in the Cd K-edge FT EXAFS data should allow for the identification of interstitial Cu impurities.

### Evidence for interstitials during cation exchange

**Fig. 3:**
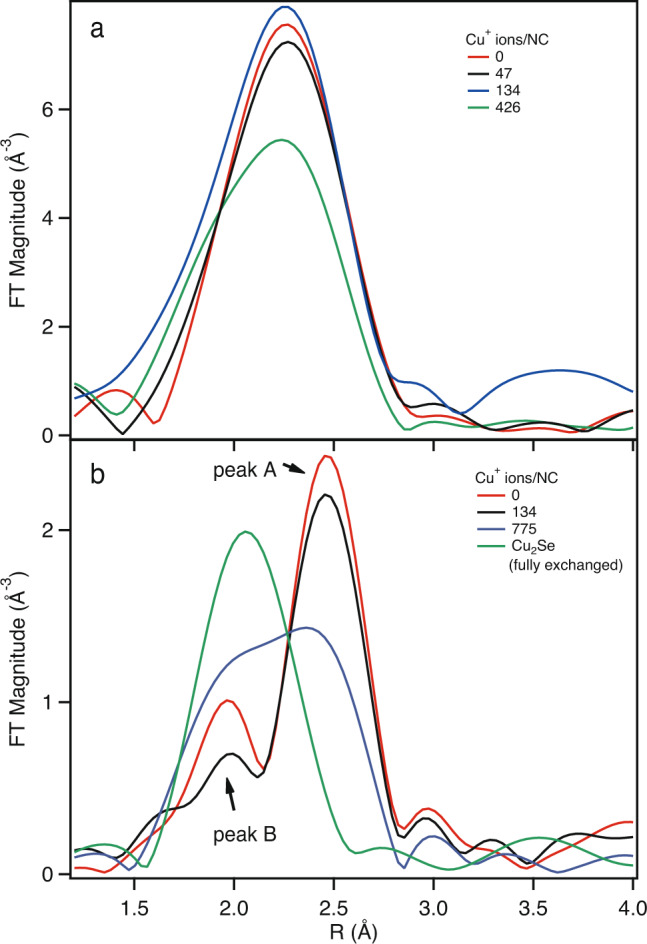
Fourier transformed EXAFS data at the Cde and Se K-edges. Magnitude of the (**a**) Cd K-edge and (**b**) Se K-edge Fourier transformed EXAFS data for 5.0 nm CdSe NCs at various Cu concentrations.

Experimental Cd K-edge FT EXAFS data is presented in Fig. 3a for 5.0 nm CdSe NCs at various stages of the cation exchange reaction. Some striking behavior is immediately observable by the eye. First, the broadening of the NN scattering peak increases with increasing Cu salt addition. Second, a shift to lower *R* is evident with increasing Cu salt content. Based upon our FEFF simulations, these observations strongly suggest the appearance of interstitial Cu atoms during the CE reaction in CdSe NCs. If the CE reaction was proceeding via a purely substitutional process, we would not expect any broadening or shifting of the NN peak; indeed, a decrease in peak amplitude would be the expectation. We would like to point out that this broadening is not a consequence of a contribution of components of Cd bonded to low-*Z* elements (i.e., Cd-O). We assert this for two reasons: (1) the Cd–O complexes that arise during this reaction occur ~1.76 Å which is ~0.15 Å lower than the expected location of the Cu interstitial (not phase shift corrected) and (2) the broadening is present even when there is no observable changes in the XANES features (i.e., when the CE process has not produced any CdO-like byproducts) [see Fig. S5].

The Se K-edge FT EXAFS data can also be used for qualitative understanding of the CE process, although the interpretation is slightly more involved. Figure 3b plots the Se K-edge FT EXAFS spectra for undoped CdSe, mol Cu/mol Cd of 0.12 and 2.9, and Cu_2_Se (i.e., fully exchanged). Immediately obvious is the strong overlap between the NN scattering peak for Cu_2_Se, the expected scattering contribution for an interstitial Cu in CdSe, and the small component of the NN scattering peak (peak A) for undoped CdSe (Fig. S6). As the Cu_2_Se NN scattering peak occurs ~0.1 Å higher than undoped CdSe peak A and the Cu interstitial peak is ~0.1 Å lower relative to the same peak, we postulate that monitoring changes in the amplitude of the CdSe peak A can provide insight into the CE process. For instance, we would expect that the introduction of the Cu salt into the CdSe solution would lead to a broadening of peak A for interstitial Cu and simply a reduction in intensity for substitutional Cu. As more Cu is introduced and the CE proceeds and eventually promotes the conversion of CdSe to Cu_2_Se, we would expect an increase in amplitude and a shifting of peak A to lower *R* towards the expected position for the main NN scattering peak in Cu_2_Se. When inspecting Fig. 3, the expected behavior is observed. For lower concentrations of Cu (mol Cu/mol Cd = 0.12; ~134 Cu atoms/NC), we observe a broadening of peak A which is consistent with interstitial Cu and the conclusions from the Cd K-edge EXAFS results. For large concentrations of Cu (mol Cu/mol Cd = 2.9; ~775 Cu atoms/NC), peak A has been replaced by a larger amplitude peak which is shifted on the order of 0.1 Å and is consistent with a co-existence of both Cu_2_Se and CdSe. The fully exchanged material is 100% Cu_2_Se and exhibits only one scattering component, as expected.

We finally note that while it is understood that for every two interstitial Cu atoms incorporated into the NC we would require one Cd vacancy, we chose to exclude the inclusion of Cd vacancies in our calculations. Even with this exclusion, the qualitative correlations between our simulations and experiment lends credence to our approach and suggest a simple mechanism of Cu inclusion followed by Cd vacancy formation and transformation into Cu_2_Se.

## Discussion

Details on the CE mechanism can be ascertained quantitatively via fitting of the EXAFS data. We first fit the starting CdSe solution (i.e., with no added Cu) to a wurtzite CdSe model and use the fitted amplitude factor (${S}_{0}^{2}$) as a set parameter for the remainder of the fits (i.e., with Cu added to the solution) to extract the coordination numbers, CN_i_. To consider the inclusion of Cu, we introduce a scattering path related to Cu interstitials (Cu_i_). Of course, during the course of the CE reaction, conversion of CdSe to Cu_2_Se leads to the formation of a Cd^2+^ byproduct, which we model in terms of a CdO scattering path for the Cd K-edge EXAFS fitting. For the Se K-edge EXAFS fitting, we include a scattering path related to Cu-Se scattering in the Cu_2_Se crystal, which is distinct from the Cu interstitial path. All of the fitting paths are treated holistically, with the rejection (inclusion) of a path during a fit resulting from unphysical fit values (an improvement of the R-factors from the fit). As an example, in Figure S7 we display fits of the Cd K-edge FT EXAFS data for 5.0 nm CdSe with 426 Cu^+^ ions/NC added. In this example, both the inclusion or exclusion of Cu_i_ lead to reasonable fits, but the inclusion of Cu_i_ results in a 40% improvement in the fit R-factors (0.006 vs. 0.01) which allows the statement that the interstitial path is present during that concentration step. All of the fitted EXAFS data can be found in the SI. For ease of the discussion, we will focus on the 5.0 nm CdSe NC, as we have done throughout the entire text.

**Fig. 4:**
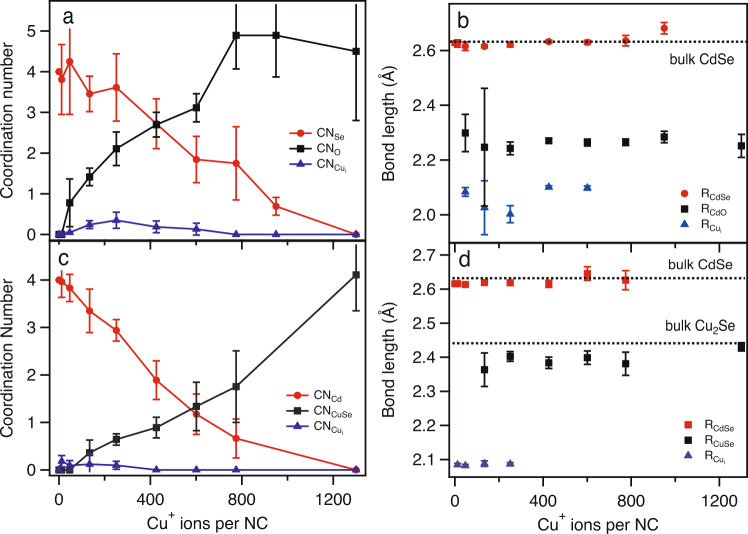
Change in coordination numbers and bond lengths as a function of copper concentration. Values of the coordination numbers and bond lengths from fits of the Cd K-edge (**a**, **b**) and Se K-edge (**c**, **d**) EXAFS data for 5.0 nm CdSe at various stages of the copper cation exchange reaction. The error bars represent uncertainties in the EXAFS fitting, and specific details on how these errors are calculated can be found in Ref. ^33^.

Figure 4 displays the coordination numbers, CN_i_, and bond lengths, *R*_i_, derived from the EXAFS fits for 5.0 nm CdSe NCs. The general behavior for the coordination numbers for the Cd K-edge (Fig. 4a) and the Se K-edge (Fig. 4c) is similar. With increasing Cu concentration, the average values for the coordination numbers for Se (Cd) for the Cd (Se) K-edge decrease and eventually are zero at a critical Cu concentration, as expected for complete conversion from CdSe → Cu_2_Se. Conversely (but expectedly), the average values of the coordination numbers for O (Cu) for the Cd (Se) K-edge increase with increasing Cu concentration. The coordination number for the Cu_i_ path increases with Cu concentration, reaches a maximum, and then decreases towards zero with increasing Cu concentration. Values for CN_O_ tend to asymptote near 5-6, which suggest some form of a five- or six-fold coordinate Cd^2+^ complex. We note that we cannot exactly quantify the state of the Cd byproduct but the derived bond lengths (*R*_CdO_ ~ 2.3 Å) support the existence of a five- or six-fold coordinate Cd species bonded to a low-*Z* element^24^. The values for *R*_CdSe_ and *R*_CuSe_ are both remarkably consistent throughout the concentration steps, again supporting the observation of a two phase process, as severe structural perturbations or the creation of new phases could lead to much larger deviations in the derived bond lengths^25^. In addition, the fitted values of ${R}_{{\text{Cu}}_{\text{i}}}$ ~ 2.1 Å which are similar values to ${R}_{{\text{Cu}}_{\text{i}}}$ that have been observed in related materials^26^.

**Fig. 5:**
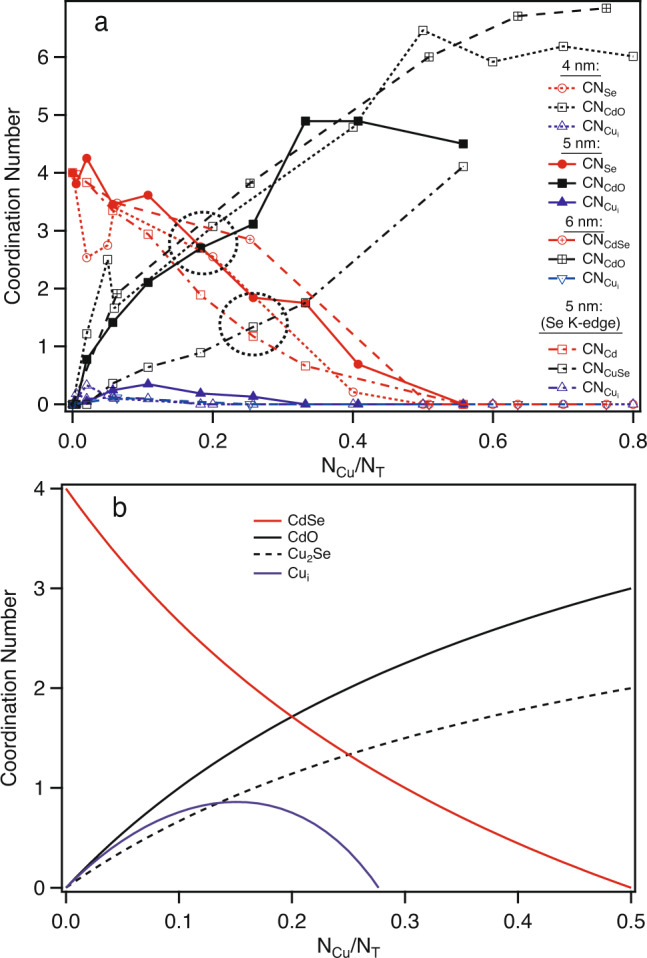
Comparison of the experimental and theoretical changes in coordination number with copper concentration. Coordination number, CN, versus the number of copper atoms (N_Cu_) normalized to the number of atoms (N_T_) in the nanocrystal. (**a**) Experimental data (CN extracted via fits of the EXAFS data) for three different particles sizes and two different metal K-edges (error bars have been removed for clarity; for an idea of the general error associated with the data points, see the error bars in Fig. 4a and c) and (**b**) Theoretically predicted change in CN with concentration. Details on the theoretical calculations are provided in the main text.

While the critical variable which controls the degree of complete cation exchange is the copper concentration, this is fundamentally an extensive property that has no transferability to another system (i.e., different sized particles). The number of copper atoms present per total atoms present in the NC ($\frac{{N}_{\text{Cu}}}{{N}_{\text{T}}}$), however, should represent an intensive property that should represent a meaningful and comparable metric to compare across various length scales. Figure 5a displays the values of CN as a function of $\frac{{N}_{\text{Cu}}}{{N}_{\text{T}}}$ derived from the fits of the Cd K-edge EXAFS data for three different particle sizes. Interestingly, when plotted against this atom normalized variable ($\frac{{N}_{\text{Cu}}}{{N}_{\text{T}}}$), the size-dependent behavior is remarkably similar. The plots for CN_Se_ and CN_O_ intersect at $\frac{{N}_{\text{Cu}}}{{N}_{\text{T}}} \sim 0.2$ irrespective of particle size. In addition, all sizes show an increase and subsequent decrease in CN$_{{\text{Cu}}_{\text{i}}}$ as a function of $\frac{{N}_{\text{Cu}}}{{N}_{\text{T}}}$.

This size-independent behavior in CN with Cu concentration speaks to a general phenomenon across length scales. We also point out that this type of behavior is consistent with observations by other groups using different probing methods. For instance, the Jain group used optical spectroscopy^7^ to probe the CE process of Cu in CdSe and found a similar crossover behavior but at a different critical concentration than we report ($\frac{{N}_{\text{Cu}}}{{N}_{\text{T}}} \sim 0.5$). We note, however, that the *y*-axis of their data is in terms of the percent of NCs that have transformed from one species to another. In their data, the crossover occurs at the point where 50% of the species have converted from CdSe to Cu_2_Se. When defined in terms of an expected CN_Se_ = 2 from a 50% mixture of CdSe and Cu_2_Se, the expected $\frac{{N}_{\text{Cu}}}{{N}_{\text{T}}} \sim$ 0.17, which matches the values we report. So, it must be noted that the exact crossover point can change depending on the specific variables being observed. As an example, we also plot $\frac{{N}_{\text{Cu}}}{{N}_{\text{T}}}$ derived from the fits of the Se K-edge EXAFS data in Fig. 5a. For the Se K-edge data for the 5.0 nm CdSe NC, the crossover point occurs $\frac{{N}_{\text{Cu}}}{{N}_{\text{T}}} \sim 0.25$. We note that we only have high quality Se K-edge EXAFS data for the 5.0 nm CdSe NCs. The ability to obtain high quality data rests on a number of variables including chemical stability (precipitation during the reaction) and absorption issues (lower *Z* elements buried in a matrix of higher *Z* elements).

The experimental data can be simply explained by considering the changes in the average coordination numbers as increased amounts of copper are introduced during the stages of the CE reaction. For comparison, we have calculated and plotted (Fig. 5b) the expected change in coordination number, CN, as a function of Cu concentration ($\frac{{N}_{\text{Cu}}}{{N}_{\text{T}}}$) based upon the following relationships (the justification for these equations can be found in Methods),

1 $${\langle \text{CN}\rangle }_{\text{CdSe}}=\frac{2-4\frac{{N}_{\text{Cu}}}{{N}_{\text{T}}}}{0.5+\frac{{N}_{\text{Cu}}}{{N}_{\text{T}}}};\,\,{\langle \text{CN}\rangle }_{\text{CdO}}=\frac{6\frac{{N}_{\text{Cu}}}{{N}_{\text{T}}}}{0.5+\frac{{N}_{\text{Cu}}}{{N}_{\text{T}}}}$$

2 $${\langle \text{CN}\rangle }_{\text{CuSe}}=\frac{4\frac{{N}_{\text{Cu}}}{{N}_{\text{T}}}}{0.5+\frac{{N}_{\text{Cu}}}{{N}_{\text{T}}}};{\langle \text{CN}\rangle }_{{\text{Cu}}_{\text{i}}}=\frac{8\frac{{N}_{\text{Cu}}}{{N}_{\text{T}}}}{0.5+\frac{{N}_{\text{Cu}}}{{N}_{\text{T}}}}+\frac{{\text{CN}}_{\text{i}}\frac{{N}_{\text{Cu}}}{{N}_{\text{T}}}}{\frac{{N}_{\text{Cu}}}{{N}_{\text{T}}}-0.5}$$

where CN_i_ is the value of the CN for interstitial copper at the critical concentration where ~50% of the CdSe species have converted to Cu_2_Se. The similarity in the behavior of the experimentally and predicted changes in CN with concentration are striking. The theoretically expected crossover point for the CdSe/CdO species occurs at $\frac{{N}_{\text{Cu}}}{{N}_{\text{T}}} \sim 0.2$ while the crossover point for the CdSe/Cu_2_Se species occurs at $\frac{{N}_{\text{Cu}}}{{N}_{\text{T}}} \sim 0.25$, as observed experimentally. We can also replicate the experimental behavior in the values for CN_i_. As a whole, we have shown that the copper cation exchange process in a series of different sized CdSe nanocrystals is explained by interstitial inclusions of Cu at the early stages followed by conversion of CdSe → Cu_2_Se, with the appearance of no other spurious phases.

## Methods

### Materials

Cadmium carbonate (CdCO_3_) (98%, Aldrich), selenium powder (Se) (100 mesh, 99.5% trace metals basis, ACROS), Tetrakis(acetonitrile)copper(I) hexafluorophosphate (97%, Aldrich), tri-n-octylphosphine oxide (TOPO) (99%, Aldrich), tri-n-octylphosphine (TOP) (90%, Aldrich), 1-octadecene (ODE) (90%, Aldrich), stearic acid (SA) (95%), toluene (99.5%, VWR), methanol (Fisher, 99.9%), and ethanol (Alfa Aesar, 94–96%). All chemicals were purchased and used without further purification.

### Synthesis of CdSe nanocrystals

CdSe nanocrystals (NCs) were synthesized via a modified hot injection method from Shakeri^19^ and Webber^20^. A typical synthesis involves stirring 3.4 mmol (590 mg) of CdCO_3_ with 5.0 g of TOPO and 5.0 g of SA in a three-neck, round bottom flask, heated to 120 °C under flowing N_2_. The solution is held isothermally for 1.5 h before heating to 360 °C. Separately, 4.9 mmol (383 mg) of Se powder is dissolved in 5.0 mL of TOP under N_2_. The Se solution is quickly injected into the reaction flask and followed by immediate injection of 5.0 mL of ODE. To quench the reaction, the flask is removed 2–5 min after the first injection or once the desired size is achieved. When the NC solution approaches 50 °C, 5 mL of toluene is injected to facilitate the purification process. Once at room temperature, the mixture is distributed into three 45-mL centrifuge tubes. Each centrifuge tube receives 5 mL of toluene, 6 mL of ethanol, and 5 mL of methanol. The mixture is shaken for 30 s and centrifuged at 3050 rpm for 5 min. The supernatant is decanted and the precipitate is purified twice. The purification process is continued by re-dispersing the NCs in 5 mL of toluene and adding 6 mL of ethanol and 5 mL of methanol to each centrifuge tube. The tubes are then shaken for 30 s and centrifuged for 5 min. The final product was dried under flowing N_2_ overnight and sealed under a N_2_ atmosphere.

### Copper cation exchange and X-ray absorption fine structure spectroscopy (XAFS)

X-ray absorption fine structure (XAFS) experiments were performed at Sector 10 on the insertion device line operated by the Materials Research Collaborative Access Team (MRCAT)^27^. Measurements were performed in both transmission and fluorescence using a Lytle detector. XAFS measurements were performed on a 0.2 M (5.2 × 10^−4^ mol) toluene solvated CdSe NC solution in a custom-built peristaltic pump-driven flow cell. Aliquots of a 0.1 M (Copper precursor) in ethanol (1.1 × 10^−3^ mol) were added to the stirred CdSe solution and allowed to equilibrate. The concentration steps are presented for completeness in the Supporting Information (Table S1). After addition of the copper salt solution, XAFS scans were taken at the Cu, Cd, and Se K-edges. Calibrations were done on Cd(NO_3_)_2_ powder, or Cu and Se foils for the Cd, Cu, and Se edges, respectively.

XAFS data processing and analysis were done using the IFEFFIT suite of programs^28^. Initial estimates of the threshold energy values (*E*_0_) were obtained via the inflection point in the normalized absorption edges. A Hanning window was applied to a selected *k*-range (nominally 3–11 Å^−1^) to obtain the Fourier transformed (FT) extended XAFS (EXAFS) data. For quantitative determination of both coordination numbers (CN_*i*_), bond lengths (*R*_*i*_), and EXAFS Debye–Waller factors (static disorders, ${\sigma }_{i}^{2}$), the experimental data was fit using CdSe, Cu_2_Se, interstitial Cu, or CdO as the fitting models. FEFF6 was used to calculate the photoelectron scattering path amplitudes, *F*_*i*_(*k*), and phase, *ϕ*(*k*), and the samples were fit to the EXAFS equation,

3 $$\chi (k)=\frac{(C{N}_{i}{S}_{o}^{2})}{(2k{R}_{i}^{2})}{F}_{i}(k){e}^{(-2{k}^{2}{\sigma }_{i}^{2})}\sin [2k{R}_{i}+{\phi }_{i}(k)].$$

### Ultra-violet Visible (UV–Vis) and Photoluminescence (PL) Spectroscopy

The UV–Vis spectra were recorded in toluene on an Ocean Optics USB2000 spectrophotometer using a 1 cm path length quartz cell. The absorbance values of the first absorption maximum were used to calculate the NC diameters using the empirical formula reported by Peng and co-workers^29^ and Mulvaney and co-workers^30^. PL spectra were measured using an excitation wavelength of *λ*_*max*_ = 405 nm (*E* = 3.06 eV) and the signal collected using an Ocean Optics USB2000 CCD spectrometer.

### X-ray Diffraction (XRD)

NC structure was studied using XRD by *θ*–2*θ* scans with Cu K*α* radiation using line focus on a PANalytical X’PertPro Diffractometer. XRD samples were prepared by drop-casting a small amount of the NC solution onto a glass slide.

### Theoretical details

To calculate the CdSe coordination number, 〈CN〉_CdSe_, we calculate,

4 $${\langle \text{CN}\rangle }_{\text{CdSe}}=\frac{{\text{CN}}_{\text{bulk}}^{\text{CdSe}\,}(n-m)}{n+m}$$

where *n* = # of Cd atoms and *m* = # of Cu atoms. We re-define *m* ≡ N_Cu_ and *n* ≡ N_Cd_ and note that N_Cd _= N_T_/2, where N_T_ is the total # of atoms in the undoped NC. We realize that this is a crude assumption on the exact stoichiometry of CdSe NCs^31,32^, but for our purposes, we find it an appropriate approximation. The above expression then becomes,

5 $${\langle \text{CN}\rangle }_{\text{CdSe}}=\frac{{\text{CN}}_{\text{bulk}}^{\text{CdSe}\,}({N}_{\text{T}}/2-{N}_{\text{Cu}})}{{N}_{\text{T}}/2+{N}_{\text{Cu}}}$$

We divide through by *N*_T_ and note that CN${\,}_{\,\text{bulk}}^{\text{CdSe}\,}=4$, we can write,

6 $${\langle \text{CN}\rangle }_{\text{CdSe}}=\frac{2-4{N}_{\text{Cu}}/{N}_{\text{T}}}{0.5+{N}_{\text{Cu}}/{N}_{\text{T}}}$$

For the CdO or Cu_2_Se coordination environments, we follow similar logic but only account for the number of CdO or Cu_2_Se that is created,

7 $${\langle \text{CN}\rangle }_{\text{CdO/CuSe}}=\frac{{\text{CN}}_{\text{bulk}}^{\text{CdO/CuSe}\,}m}{n+m}$$

Using CN${\,}_{\,\text{bulk}}^{\text{CdO}\,}=6$ and CN${\,}_{\,\text{bulk}}^{\text{CuSe}\,}=4$, we arrive at,

8 $${\langle \text{CN}\rangle }_{\text{CdO}}=\frac{6\frac{{N}_{\text{Cu}}}{{N}_{\text{T}}}}{0.5+\frac{{N}_{\text{Cu}}}{{N}_{\text{T}}}}$$

9 $${\langle \text{CN}\rangle }_{\text{CuSe}}=\frac{4\frac{{N}_{\text{Cu}}}{{N}_{\text{T}}}}{0.5+\frac{{N}_{\text{Cu}}}{{N}_{\text{T}}}}$$

To derive the equation for interstitial Cu, we note that we expect the number to Cu interstitials to increase with concentration up to a critical point denote by some coordination number CN_i_, at which there is enough Cu present to form Cu_2_Se and therefore results in a decrease in Cu interstitial concentration. We can expect, then, that such a dependence can be written as,

10 $${\langle \text{CN}\rangle }_{{\text{Cu}}_{\text{i}}}=\frac{{\text{CN}}_{\text{bulk}}^{\text{i}}m}{n+m}+\frac{{\text{CN}}_{\text{i}}m}{n+m-2m}$$

11 $${\langle \text{CN}\rangle }_{{\text{Cu}}_{\text{i}}}=\frac{{\text{CN}}_{\text{bulk}}^{\text{i}}m}{n+m}+\frac{{\text{CN}}_{\text{i}}m}{n-m}$$

We note that the term (-2*m*) appears in the denominator because due to the stoichiometry of the CE reaction. Using similar logic as above, we can re-write this expression as,

12 $${\langle \text{CN}\rangle }_{{\text{Cu}}_{\text{i}}}=\frac{8\frac{{N}_{\text{Cu}}}{{N}_{\text{T}}}}{0.5+\frac{{N}_{\text{Cu}}}{{N}_{\text{T}}}}+\frac{{\text{CN}}_{\text{i}}\frac{{N}_{\text{Cu}}}{{N}_{\text{T}}}}{\frac{{N}_{\text{Cu}}}{{N}_{\text{T}}}-0.5}$$

where CN_i_ is the value of the CN for interstitial copper at the critical concentration where ~ 50% of the CdSe species have converted to Cu_2_Se.

## Supplementary information

Supplementary Information

Peer Review File

## Data Availability

The data that support the findings of this study are available from the corresponding author upon reasonable request.
