# Supplementary material for: Mechanistic insight into copper cation exchange in cadmium selenide semiconductor nanocrystals using X-ray absorption spectroscopy
Source: Nat Commun. 2021 Jan 19;12:438. doi: 10.1038/s41467-020-20712-0 (PMC7815718; doi:10.1038/s41467-020-20712-0)
Supplement: Supplementary file 1 — Supplementary Information [file 41467_2020_20712_MOESM1_ESM.pdf]

Supplemental Information for

Mechanistic Insight into Copper Cation Exchange in Cadmium Selenide Semiconductor Nanocrystals using X-ray Absorption Spectroscopy

Alex Khammang,[1] Joshua T. Wright,[2] and Robert W. Meulenberg[1],

[1]Department of Physics and Astronomy and Frontier Institute for Research in Sensor Technologies, University of Maine, Orono, ME 04469

[2]Department of Physics, Illinois Institute of Technology, Chicago IL

Contents

Figure S1. Cd K-edge XANES spectra for 4.0 and 6.0 nm CdSe NCs at different Cu concentrations............2
Figure S2. Se K-edge XANES spectra for 4.0 and 6.0 nm CdSe NCs at different Cu concentrations.............2
Figure S3. Cu K-edge XANES spectra for 4.0 and 6.0 nm CdSe NCs at different Cu concentrations............3
Figure S4. Cd K-edge, Se K-edge, and Cu K-edge XANES spectra for 3.0 nm CdSe NCs at different Cu concentrations..3
Figure S5. Cd K-edge, Se K-edge, and Cu K-edge XANES spectra for 5.0 nm CdSe NCs at low Cu concentrations..4
Figure S6. Path contributions calculated from FEFF9 for CdSe, Cu_2Se, and interstitial Cu..........................4
Figure S7. Magnitude of the FT EXAFS (red dots) with fits (black lines) for 5.0 nm CdSe NCs considering both interstitial and no interstitial Cu paths during fitting..5
Figure S8. Cd K-edge EXAFS and FT EXAFS spectra for 4.0 nm CdSe NCs at different Cu concentrations..5
Figure S9. Cd K-edge EXAFS and FT EXAFS spectra for 5.0 nm CdSe NCs at different Cu concentrations..6
Figure S10. Se K-edge EXAFS and FT EXAFS spectra for 5.0 nm CdSe NCs at different Cu concentrations..6
Figure S11. Cd K-edge EXAFS and FT EXAFS spectra for 6.0 nm CdSe NCs at different Cu concentrations..7
Figure S12. Cd K-edge (a) EXAFS and (b) FT EXAFS spectra for 5.0 nm CdSe NCs at different Ag concentrations..7
Figure S13. Ag K-edge (a) EXAFS and (b) FT EXAFS spectra for 5.0 nm CdSe NCs at different Ag concentrations..8
Figure S14. UV-Visible and photoluminescence spectra for a series of CdSe NCs used in these studies....9
Figure S15. Changes in powder XRD for copper cation exchange in 5.0 nm CdSe for various copper concentrations..9
Table S1. Copper concentrations used during the various stages of the cation exchange reaction..........10
Table S2. Cd K-edge EXAFS fitting results for 4.0 nm CdSe NCs..11
Table S3. Cd K-edge EXAFS fitting results for 5.0 nm CdSe NCs..12
Table S4. Se K-edge EXAFS fitting results for 5.0 nm CdSe NCs...13
Table S5. Cd K-edge EXAFS fitting results for 6.0 nm CdSe NCs..14

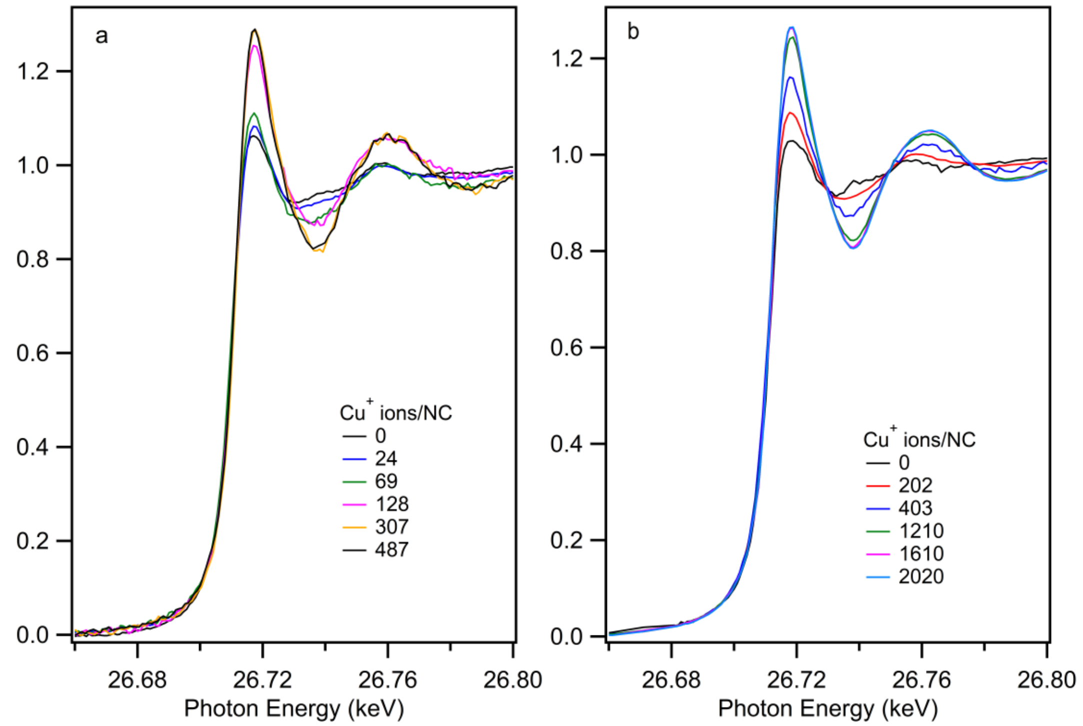

Figure S1. Cd K-edge XANES spectra for (a) 4.0 and (b) 6.0 nm CdSe NCs at different Cu concentrations.

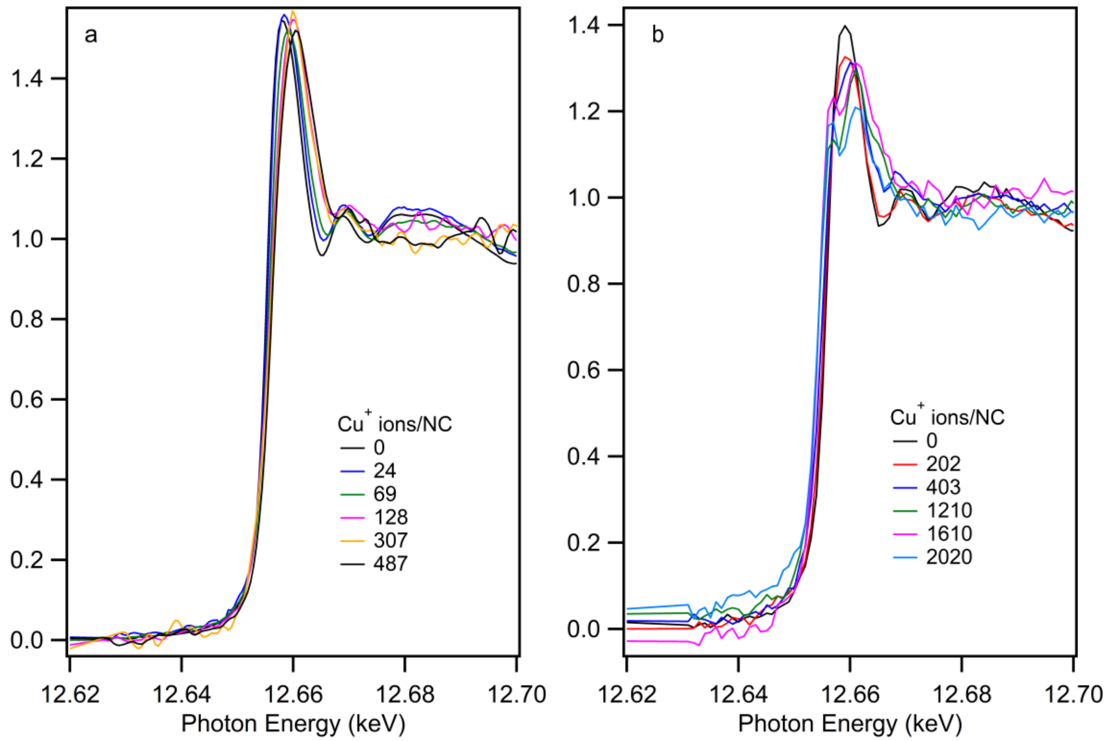

Figure S2. Se K-edge XANES spectra for (a) 4.0 and (b) 6.0 nm CdSe NCs at different Cu concentrations.

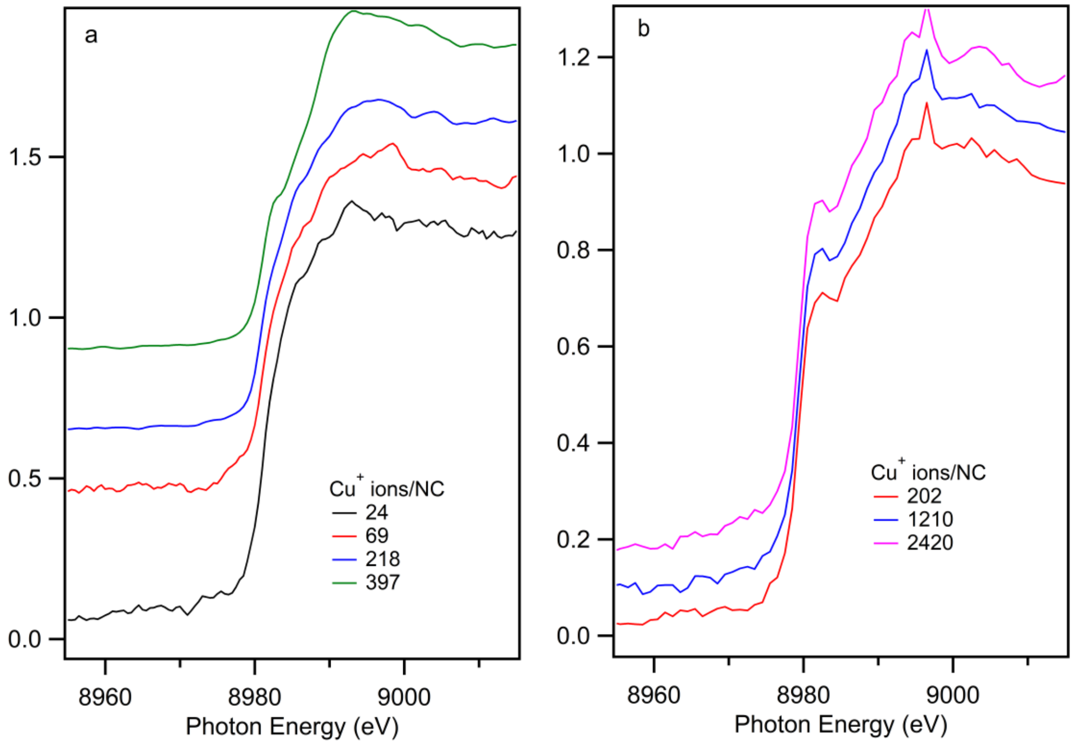

Figure S3. Cu K-edge XANES spectra for (a) 4.0 and (b) 6.0 nm CdSe NCs at different Cu concentrations.

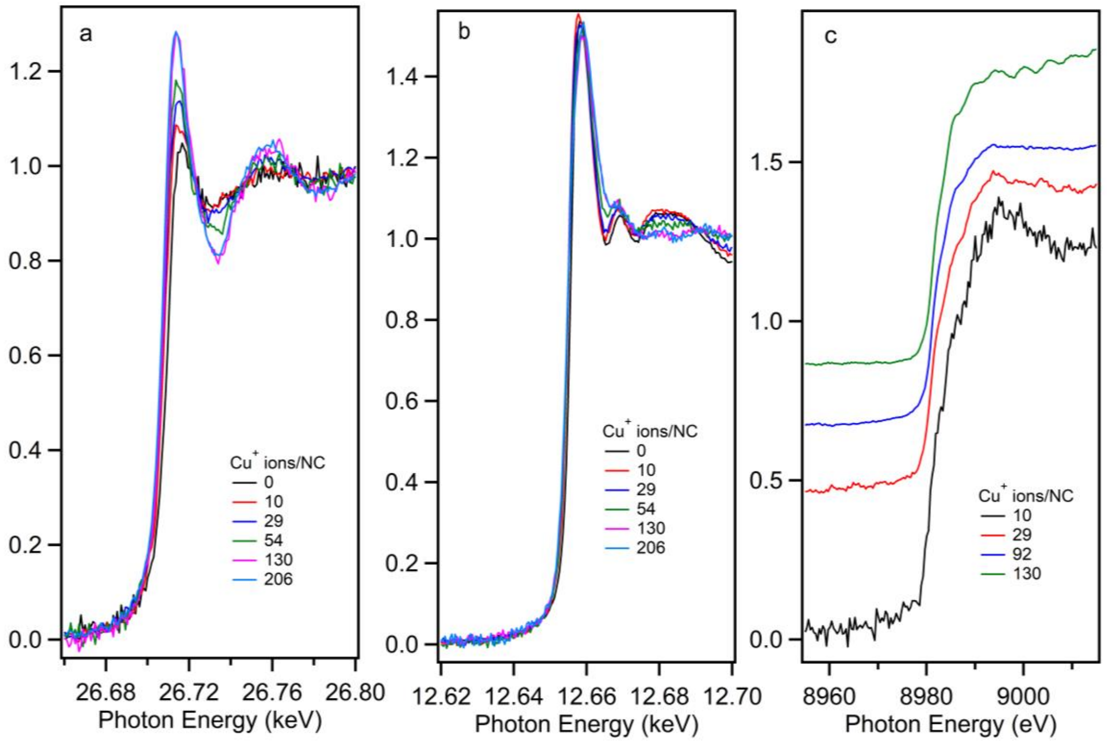

Figure S4. (a) Cd K-edge, (b) Se K-edge, and (c) Cu K-edge XANES spectra for 3.0 nm CdSe NCs at different Cu concentrations.

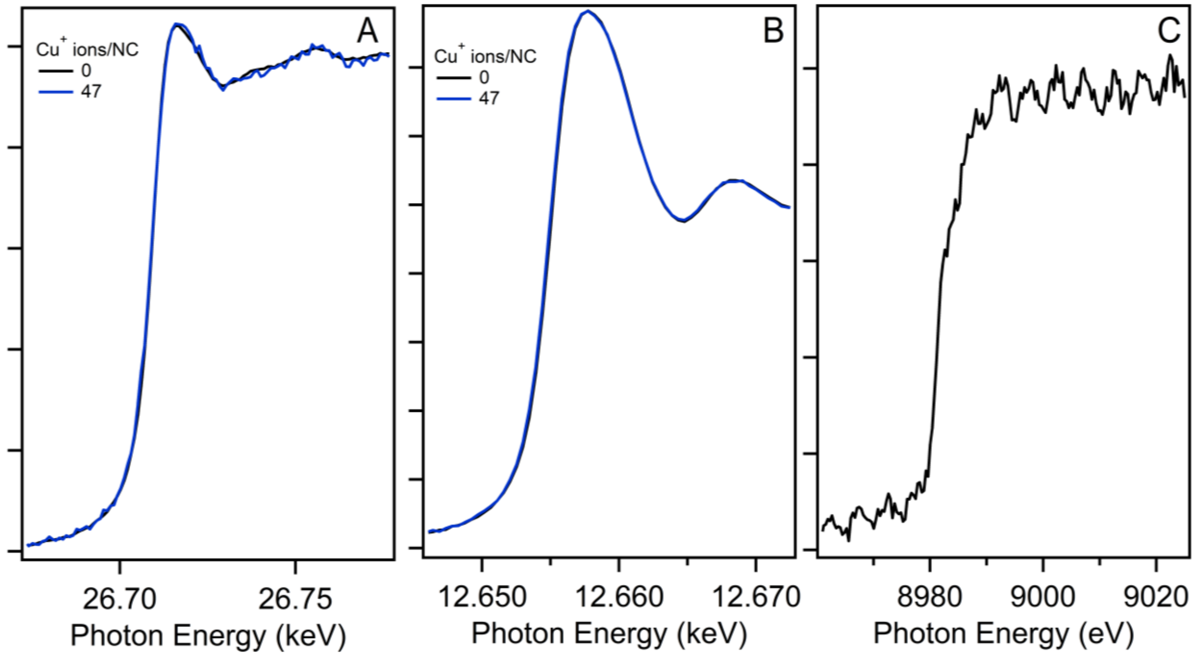

Figure S5. (a) Cd K-edge, (b) Se K-edge, and (c) Cu K-edge XANES spectra for 5.0 nm CdSe NCs at low Cu concentrations.

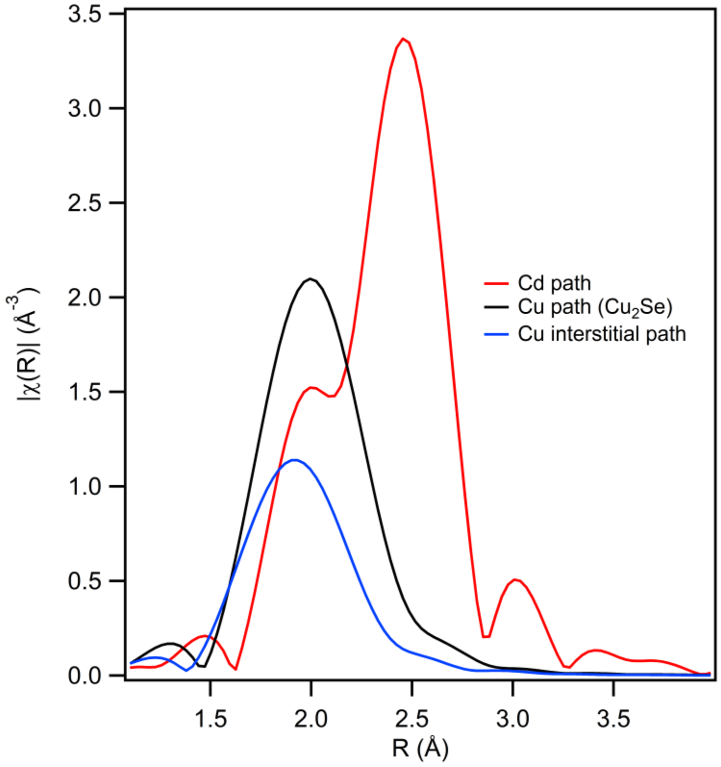

Figure S6. Path contributions calculated from FEFF9 for CdSe, Cu_2Se, and interstitial Cu in CdSe.

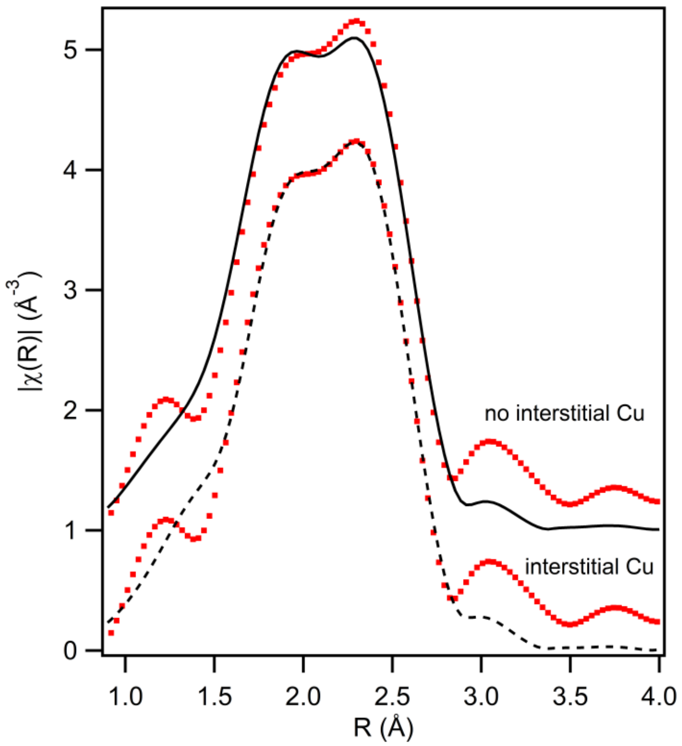

Figure S7. Magnitude of the FT EXAFS (red dots) with fits (black lines) for 5.0 nm CdSe NCs considering both interstitial and no interstitial Cu paths during fitting.

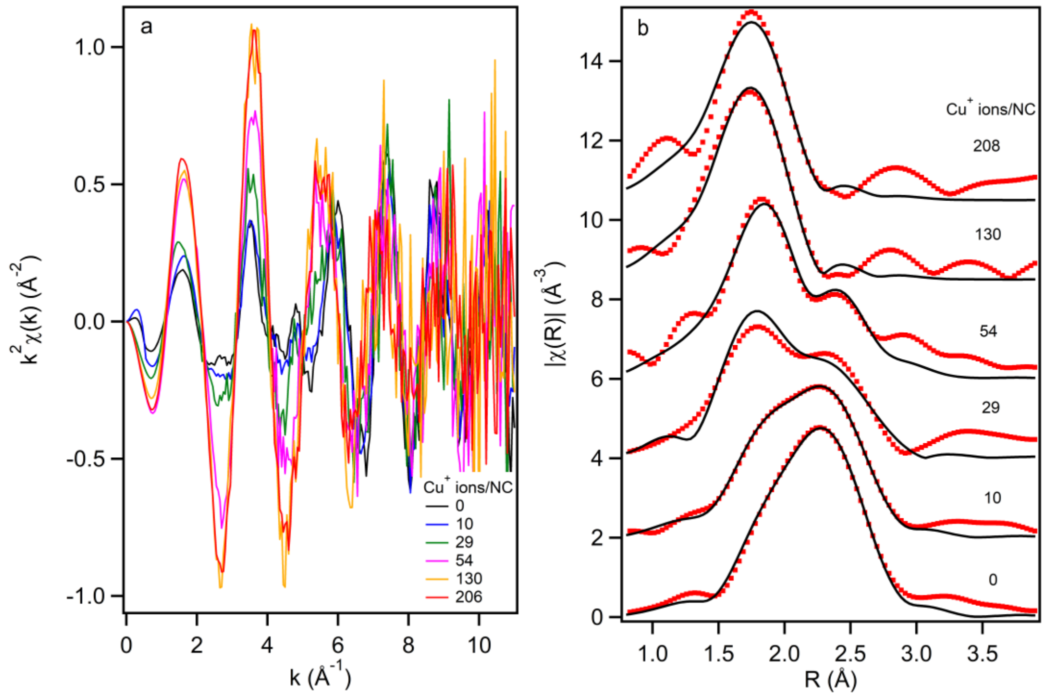

Figure S8. Cd K-edge (a) EXAFS and (b) FT EXAFS spectra for 4.0 nm CdSe NCs at different Cu concentrations. In (b) the red dots are the data and the black lines are the fits.

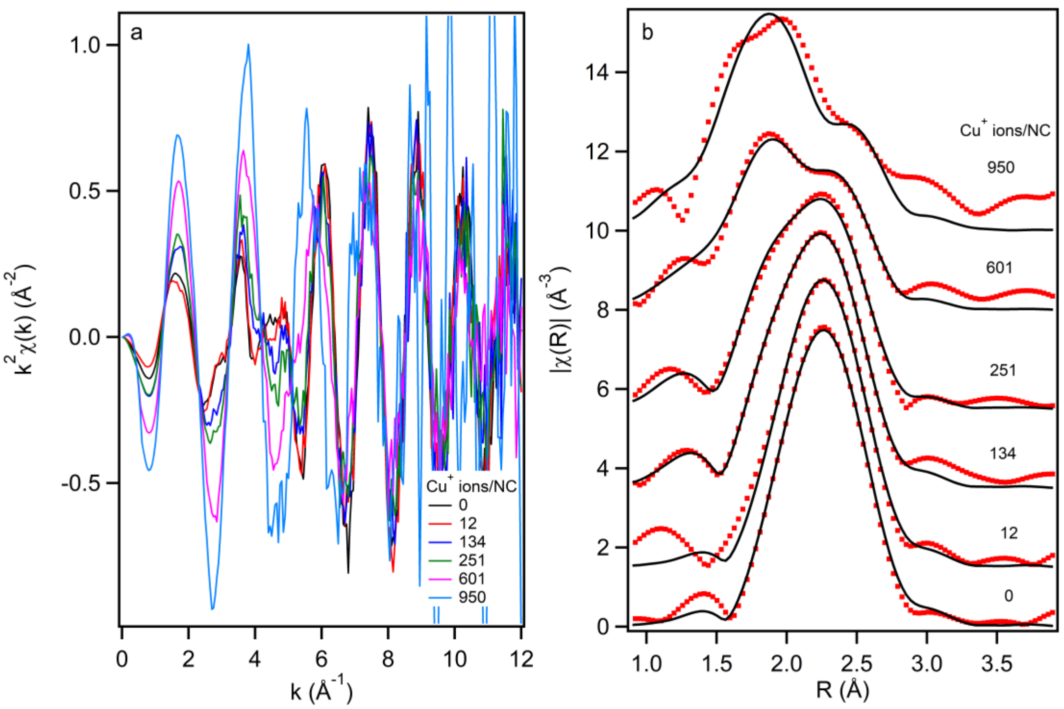

Figure S9. Cd K-edge (a) EXAFS and (b) FT EXAFS spectra for 5.0 nm CdSe NCs at different Cu concentrations. In (b) the red dots are the data and the black lines are the fits.

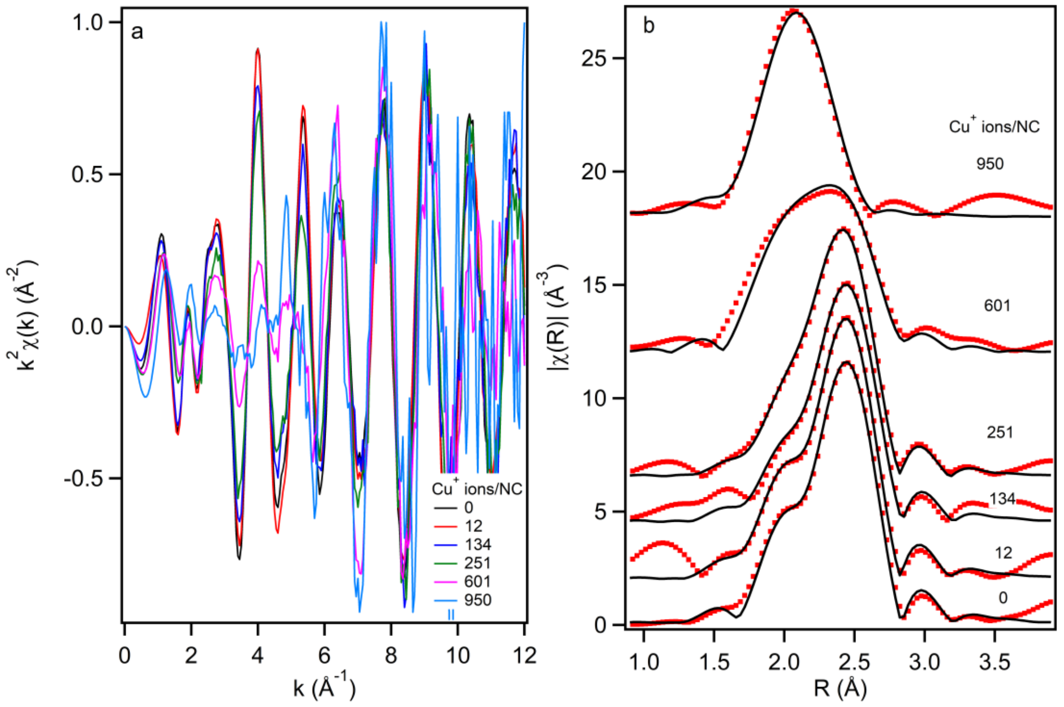

Figure S10. Se K-edge (a) EXAFS and (b) FT EXAFS spectra for 5.0 nm CdSe NCs at different Cu concentrations. In (b) the red dots are the data and the black lines are the fits.

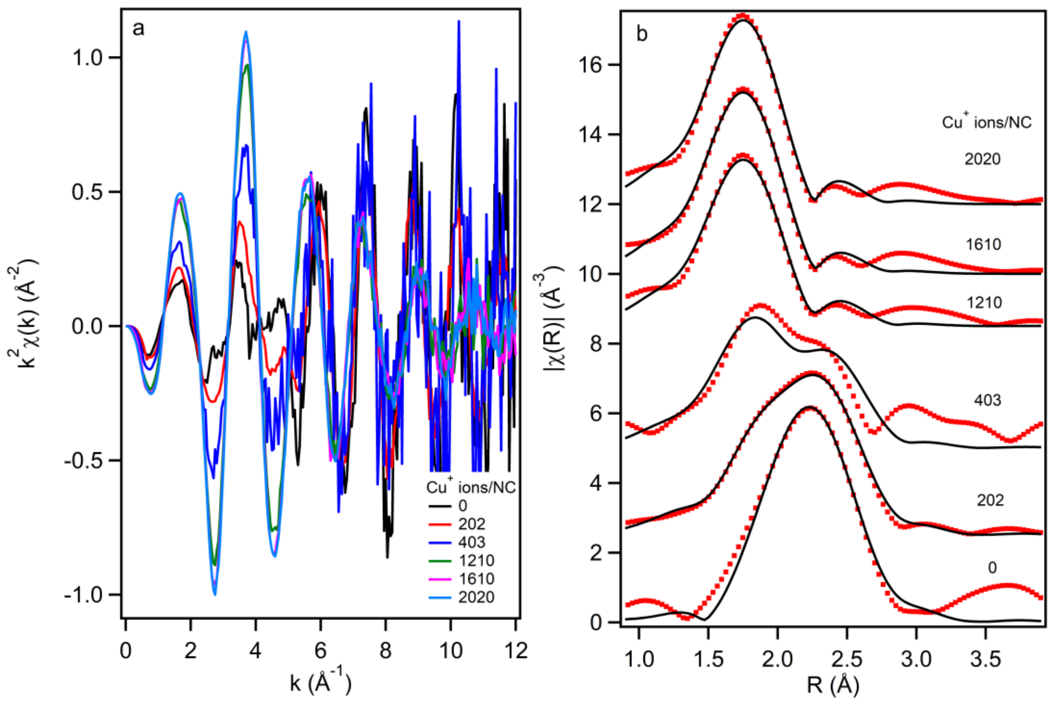

Figure S11. Cd K-edge (a) EXAFS and (b) FT EXAFS spectra for 6.0 nm CdSe NCs at different Cu concentrations. In (b) the red dots are the data and the black lines are the fits.

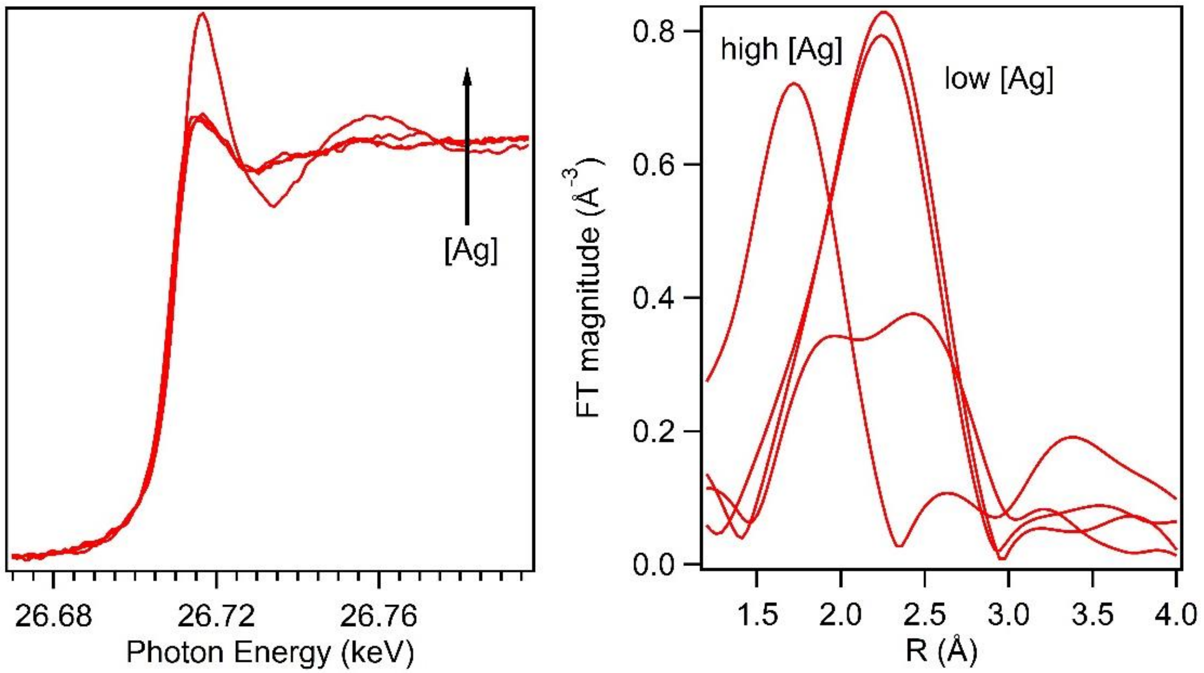

Figure S12. Cd K-edge (a) EXAFS and (b) FT EXAFS spectra for 5.0 nm CdSe NCs at different Ag concentrations.

The Cd K-edge XANES data shows the same trends with increasing Ag concentration as observed with increasing copper concentration: an increase in the XANES "white line" along with isosbestic points. The Cd K–edge EXAFS data also shows the qualitative trends we discussed in the main text of the paper: a slight reduction and broadening of the nearest neighbor scattering contribution. At moderate Ag concentrations, we observe increased broadening, and at the highest Ag concentrations (i.e. conversation to Ag_2Se), we see a peak at low *R* which is suggestive of the Cd complex that is formed upon complete exchange.

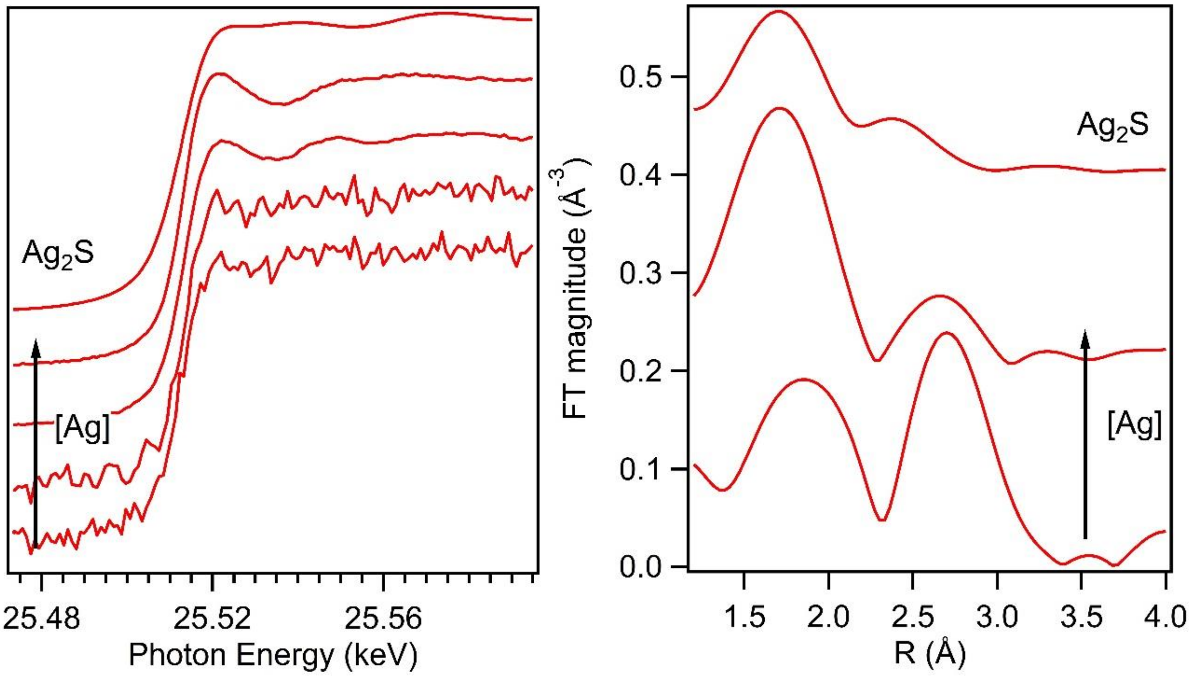

Figure S13. Ag K-edge (a) EXAFS and (b) FT EXAFS spectra for 5.0 nm CdSe NCs at different Ag concentrations.

The Ag K-edge XANES data shows a similar trend to what we observed for the Cu K-edge XANES data. We see similar spectra features throughout the Ag concentration ranges, and the data look qualitatively similar to Ag_2S (we did not have a Ag_2Se standard, and have not yet been able to return to the synchrotron). In a more exciting manner, we were able to get decent quality EXAFS data for the Sg K-edge (which we unable to do with the Cu K-edge). The data is interesting as it shows a scattering component at ~2.8 Å (phase shift correct) at low Ag concentration for high Ag concentrations, we observe a prominent scattering component at ~2.1 Å. The EXAFS is qualitatively similar to the EXFAS for Ag_2S, but we have not performed any fits on the data at this time due to the incomplete nature of the data, and the fact the all relevant controls have not been able to be performed (for instance, the scattering feature at ~2.1 Å indeed related to Ag or

perhaps related to some other spurious Ag species that competes with Ag_2Se formation as colloidal stability of the converted species decreases which means we have more difficulties getting good signal at larger concentrations).

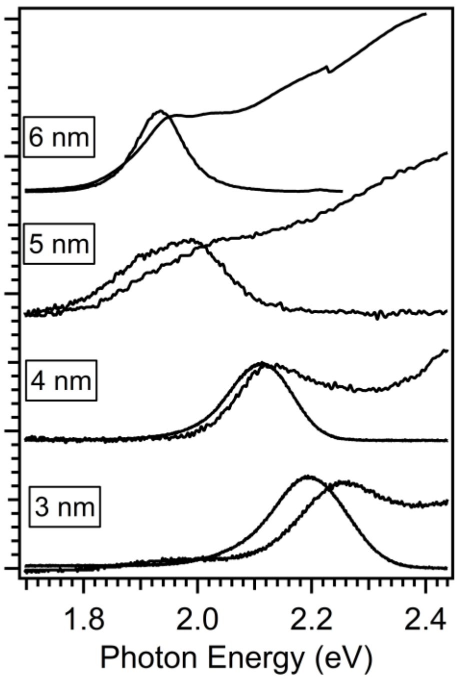

Figure S14. UV-Visible and photoluminescence spectra for a series of CdSe NCs used in these studies.

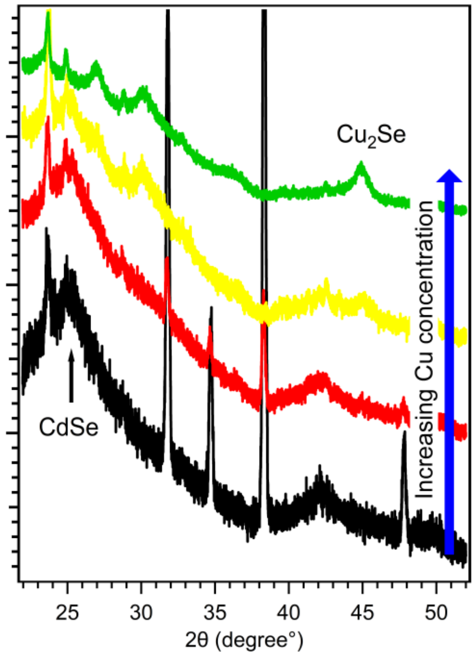

Figure S15. Changes in powder XRD for copper cation exchange in 5.0 nm CdSe for various copper concentrations.

Table S1. Copper concentrations used during the various stages of the cation exchange reaction.

Reaction step	Cu^+ added (mol)	Cu^+ ions/NC (6 nm)	Cu^+ ions/NC (5 nm)	Cu^+ ions/NC (4 nm)	Cu^+ ions/NC (3 nm)	Cu^+ ions/NC atoms
0	0	0	0	0	0	0.00
1	5.20E-06	-	12	6	3	0.01
2	1.60E-05	-	47	24	10	0.02
3	4.00E-05	202	134	69	29	0.06
4	5.00E-05	403	251	128	54	0.11
5	7.80E-05	1210	426	218	92	0.19
6	7.80E-05	-	601	307	130	0.26
7	7.80E-05	-	775	397	168	0.34
8	7.80E-05	1610	950	487	206	0.41
9	7.80E-05	2020	1125	576	243	0.49
10	7.80E-05	2420	1300	666	281	0.57
11	7.80E-05	-	1475	756	319	0.64
12	7.80E-05	2820	1650	845	357	0.72
13	7.80E-05	3220	1825	934	394	0.79
14	7.80E-05	3630	2000	1024	432	0.87
15	7.80E-05	-	2175	1113	470	0.95
16	7.80E-05	4003	2350	1203	507	1.02

Table S2. Cd K-edge EXAFS fitting results for 4.0 nm CdSe NCs.

Reaction Step	Path	[a]N	[b]R (Å)	[c]σ^2 (Å^2)	[d]ΔE (eV)	[e]R-factor
0	Cd-Se	4	2.62(2)	0.007(2)	2.7(3.0)	0.02
1	Cd-Se<br>Cd-Cu<br>Cd-O	[f]2.5(9)<br>0.3(2)<br>2.5(1.7)	2.62(2)<br>2.09(2)<br>2.26(3)	0.005(3)	3.6(3.0)	0.02
2	Cd-Se<br>Cd-Cu<br>Cd-O	2.7(1.0)<br>0.01(1)<br>3.3(5)	2.626(9)<br>2.081(9)<br>2.27(1)	0.009(3)	5.0(1.2)	0.004
4	Cd-Se<br>Cd-O	0.21(60)<br>4.8(1.4)	2.60(4)<br>2.28(3)	0.006(5)	6.6(2.9)	0.02
5	Cd-O	5.0(1.2)	2.26(1)	0.010(02)	5.0(1.2)	0.02
6	Cd-O	5.9(7)	2.270(8)	0.009(2)	5.5(6)	0.02
7	Cd-O	5.3(1.1)	2.27(1)	0.009(2)	5.4(1.1)	0.02
8	Cd-O	5.1(1.0)	2.27(1)	0.008(2)	5.1(1.0)	0.01
9	Cd-O	5.4(1.0)	2.27(1)	0.010(2)	6.6(7)	0.01
10	Cd-O	6.5(6)	2.27(1)	0.009(2)	5.2(9)	0.01
	[a]N, coordination number; [b]R, distance between absorber and backscatter atoms; [c]σ^2, Debye–Waller factor to account for both thermal and structural disorders; [d]ΔE, inner potential correction; [e]R factor, indicates the goodness of the fit; [f]S_o^2=0.93 for all the future fits					

Table S3. Cd K-edge EXAFS fitting results for 5.0 nm CdSe NCs.

Reaction Step	Path	[a]N	[b]R (Å)	[c]σ^2 (Å^2)	[d]ΔE (eV)	[e]R-factor
0	Cd-Se	4	2.627(8)	0.007(1)	7.1(1.4)	0.01
1	Cd-Se	[f]3.8(9)	2.63(1)	0.007(2)	6.6(2.4)	0.02
2	Cd-Se<br>Cd-Cu<br>Cd-O	3.96(91)<br>0.05(1)<br>0.7(1)	2.62(2)<br>2.08(2)<br>2.30(7)	0.007(2)	4.5(3.0)	0.01
3	Cd-Se<br>Cd-Cu<br>Cd-O	3.5(4)<br>0.24(9)<br>1.4(2)	2.615(9)<br>2.03(5)<br>2.25(2)	0.0074(9)	5.1(1.0)	0.002
4	Cd-Se<br>Cd-Cu<br>Cd-O	3.6(8)<br>0.35(2)<br>2.1(4)	2.622(9)<br>2.002(8)<br>2.24(2)	0.009(2)	5.3(1.6)	0.005
5	Cd-Se<br>Cd-Cu<br>Cd-O	2.7(6)<br>0.19(15)<br>2.7(3)	2.633(5)<br>2.101(5)<br>2.271(9)	0.008(2)	6.9(3)	0.006
6	Cd-Se<br>Cd-Cu<br>Cd-O	1.8(6)<br>0.13(11)<br>3.1(3)	2.630(8)<br>2.098(8)<br>2.26(1)	0.007(2)	6.9(1.0)	0.006
7	Cd-Se<br>Cd-O	0.91(89)<br>5.3(1.2)	2.63(2)<br>2.25(3)	0.012(4)	5.7(2.0)	0.02
8	Cd-Se<br>Cd-O	0.6(2)<br>4.9(1.0)	2.68(2)<br>2.28(2)	0.007(4)	8.2(1.9)	0.03

[a]N, coordination number; [b]R, distance between absorber and backscatter atoms; [c]σ^2, Debye–Waller factor to account for both thermal and structural disorders; [d]ΔE, inner potential correction; [e]R factor, indicates the goodness of the fit; [f]S_o^2=1.1 for all the future fits

Table S4. Se K-edge EXAFS fitting results for 5.0 nm CdSe NCs.

Reaction Step	Path	[a]N	[b]R (Å)	[c]σ^2 (Å^2)	[d]ΔE (eV)	[e]R-factor
0	Se-Cd	4	2.616(5)	0.0055(4)	2.2(4)	0.004
1	Se-Cd<br>Se-Cu_i	[f]3.96(32)<br>0.18(10)	2.616(4)<br>2.084(4)	0.0056(6)	2.3(6)	0.01
2	Se-Cd<br>Se-Cu_i	3.83(3)<br>0.09(08)	2.613(4)<br>2.082(4)	0.0055(5)	1.9(5)	0.01
3	Se-Cd<br>Se-Cu_i<br>Se-Cu	3.3(5)<br>0.12(9)<br>0.36(27)	2.620(8)<br>2.088(8)<br>2.36(27)	0.005(1)	2.4(1.0)	0.007
4	Se-Cd<br>Se-Cu_i<br>Se-Cu	2.9(2)<br>0.09(8)<br>0.6(1)	2.619(9)<br>2.087(8)<br>2.40(2)	0.0044(6)	2.5(5)	0.003
5	Se-Cd<br>Se-Cu	1.8(4)<br>0.9(2)	2.62(1)<br>2.38(2)	0.003(1)	1.5(1.6)	0.03
6	Se-Cd<br>Se-Cu	1.2(4)<br>1.3(5)	2.64(2)<br>2.40(2)	0.001(1)	3.1(3.0)	0.01
7	Se-Cu	1.8(8)	2.38(3)	0.003(3)	-1.4(5.5)	0.03
8	Se-Cu	4.1(8)	2.43(1)	0.008(1)	2.0(2.0)	0.02
	[a]N, coordination number; [b]R, distance between absorber and backscatter atoms; [c]σ^2, Debye–Waller factor to account for both thermal and structural disorders; [d]ΔE, inner potential correction; [e]R factor, indicates the goodness of the fit; [f]S_o^2=0.81 for all the future fits					

Table S5. Cd K-edge EXAFS fitting results for 6.0 nm CdSe NCs.

Reaction Step	Path	[a]N	[b]R (Å)	[c]σ^2 (Å^2)	[d]ΔE (eV)	[e]R-factor
0	Cd-Se	4	2.61(1)	0.007(2)	3.1(2.3)	0.02
3	Cd-Se<br>Cd-Cu<br>Cd-O	[f]3.5(6)<br>0.11(10)<br>1.9(2)	2.63(1)<br>2.10(1)<br>2.26(1)	0.008(1)	4.3(1.0)	0.02
4	Cd-Se<br>Cd-O	1.3(7)<br>3.8(1.1)	2.62(1)<br>2.26(3)	0.003(3)	4.8(2.4)	0.02
5	Cd-O	6.0(6)	2.261)	0.008(2)	5.7(9)	0.01
8	Cd-O	6.7(5)	2.268(7)	0.009(1)	5.9(7)	0.007
9	Cd-O	6.8(5)	2.270(8)	0.009(1)	5.9(7)	0.008
	[a]N, coordination number; [b]R, distance between absorber and backscatter atoms; [c]σ^2, Debye–Waller factor to account for both thermal and structural disorders; [d]ΔE, inner potential correction; [e]R factor, indicates the goodness of the fit; [f]S_o^2=0.95 for all the future fits					
