# Supplementary material for: Mechanistic insight into copper cation exchange in cadmium selenide semiconductor nanocrystals using X-ray absorption spectroscopy
Source: Nat Commun. 2021 Jan 19;12:438. doi: 10.1038/s41467-020-20712-0 (PMC7815718; doi:10.1038/s41467-020-20712-0)
Supplement: Supplementary file 2 — Peer Review File [file 41467_2020_20712_MOESM2_ESM.pdf]

Reviewer #1 (Remarks to the Author):

The authors report in situ synchrotron-based spectroscopic studies of Cu cation exchange in colloidal CdSe nanocrystals. Cation exchange, especially this system, is well established but has been largely studied by optical and microscopy techniques that are insensitive to dopant speciation (i.e., interstitial vs substitutional). This manuscript tries to shed light on this by XANES and XAFS spectroscopy at the Cd, Se, and Cu edges using synchrotron sources with a custom-built sample holder to make observations in situ. The major conclusion of the work is that Cu+ ions enter the lattice by diffusion in interstitial sites. The data appear presented in the main text and supporting information clearly support the claims provided by the authors but there are some details that require some explanation. Regardless, these are fairly minor points and this work is expected to impact the field by demonstrating the utility of core-level spectroscopy to provide mechanistic insight into cation exchange reactions.

Criticisms and Questions:
* The synthetic details describe the reaction solution being substoichiometric with respect to Cd. It would be nice to know what the Cd:Se ratio of the starting CdSe NCs as this data would provide a better picture of the system before cation exchange. Is it possible that the availability of surface Se sites could be just as critical to the cation exchange process as hard/soft acid/base theory?

* How is the charge balanced with substitutional and interstitial Cu atoms in the FEFF simulations? Is the interstitial Cu atom actually Cu(0)? Are there Cd vacancies?

* How much of the Cd K-edge linewidth comes from inhomogeneous broadening from the ensemble?

* In the 'fully exchanged' Cu2Se Se K-edge XAFS spectrum shown in Figure 3b appears equivalent to the spectrum shown in Figure S10b with 950 Cu+ ions per NC. The corresponding Cd K-edge XAFS spectrum for this sample is shown in Figure S9b and does not appear to indicate CdSe nanocrystals are present, however, it is worthwhile trying to analyze the speciation of this presumably molecular Cd2+ byproduct. Have the authors tried to elucidate the identity of this Cd2+ species?

Minor:
Please add the average size of the starting CdSe nanocrystals in Figure S13.

Reviewer #2 (Remarks to the Author):

The manuscript presented by Khammang, Wright and Meulenberg reports a study of Cd2+-to-Cu+ cation exchange in CdSe nanocrystals by means of X-ray absorption spectroscopy techniques. Based on the results of the experiments the authors conclude that the reaction goes through interstitial inclusions of the guest cations in the host crystal lattice, one of the two debated mechanisms proposed to explain cation exchange in nanomaterials. The authors properly support and convincible proof their conclusions by carefully analyzing the experimental data. Nevertheless, in my opinion, this work does not have a potential to attract a broad interest of a research community reading the journal. First of all, in its current state the work is too specialized being focused solely on the analysis of (E)XAFS/XANES data. Second, the authors study only one certain system (i.e. Cu+-to-Cd2+ exchange), thus the results cannot be considered as general and applicable to other guest-host cations couples, especially, when nanocrystals possess a significant number of vacancies through which the diffusion of the both species can occur. Thus, this system is basically not representative. In addition, it has a questionable future in terms of practical application, because 1) the use of CdSe NCs is significantly restricted (especially in Europe) and 2) such conversion from CdSe to Cu2Se does not make much sense since to date many synthetic protocols are developed directly providing Cu2-xSe NCs of excellent quality without the need for an additional cation exchange step. Third, as the authors correctly admit, cation exchange reactions depend on many parameters. Hence, even the same guest-

host cations couple might behave differently depending on certain conditions, the nature of precursors, solvents, ligands, temperature, etc. To properly elucidate the mechanism of the exchange, several parameters should be considered and varied.

Apart from these major issues, there are some minor points which lower the quality of the work. Among them are the following.
1. The language of the manuscript requires additional proof-reading and polishing. Buttery butter constructions like "The first report of CE in NCs reported…", "Initially suggested by the Alivisatos group[6], this model suggests …", "…these observations strongly suggest the observation …", etc. do not make the text more attractive.
2. In the introduction the authors state that "The driving force (energetically) behind CE processes can be explained by considering hard/soft acid/base (HSAB) theory." This, in fact, only one of many parameters (summarized e.g. in Chem. Soc. Rev. 2013, 42, 89-96 and Chem. Rev. 2016, 116, 10852-10887), both thermodynamic and kinetic, affecting cation exchange. It is not entirely correct attributing the driving force to only one factor.
3. The formula used by the authors to calculate concentration of CdSe NCs based on optical properties (from Chem. Mater. 2003, 15, 2854-2860) was reexamined and corrected later by the group of Mulvaney (J. Phys. Chem. C 2009, 113, 19468–19474). It is recommended to use this latest version for the corresponding calculations, which in turn may quantitatively change conclusions in this work.
4. An image illustrating the mechanism of the exchange would be very useful to understand the process.

Overall, I find this work better suitable to a more specialized journal, such as e.g. Inorg. Chem. or Cryst. Growth Des., where it will find its audience.

RESPONSE TO REVIEW COMMENTS

Reviewer #1 (Remarks to the Author):

The authors report in situ synchrotron-based spectroscopic studies of Cu cation exchange in colloidal CdSe nanocrystals. Cation exchange, especially this system, is well established but has been largely studied by optical and microscopy techniques that are insensitive to dopant speciation (i.e., interstitial vs substitutional). This manuscript tries to shed light on this by XANES and XAFS spectroscopy at the Cd, Se, and Cu edges using synchrotron sources with a custom-built sample holder to make observations in situ. The major conclusion of the work is that Cu+ ions enter the lattice by diffusion in interstitial sites. The data appear presented in the main text and supporting information clearly support the claims provided by the authors but there are some details that require some explanation. Regardless, these are fairly minor points and this work is expected to impact the field by demonstrating the utility of core-level spectroscopy to provide mechanistic insight into cation exchange reactions.

Author's reply: We thank the reviewer for their positive comment which suggest our work is of high impact and is suitable for Nature Communications.

Criticisms and Questions:

** The synthetic details describe the reaction solution being substoichiometric with respect to Cd. It would be nice to know what the Cd:Se ratio of the starting CdSe NCs as this data would provide a better picture of the system before cation exchange. Is it possible that the availability of surface Se sites could be just as critical to the cation exchange process as hard/soft acid/base theory?*

Author's reply: We thank the reviewer for their comment and agree that knowledge of the stoichiometry of the starting NCs is important to know. We did perform ICP-MS of some the starting materials: Cd/Se ratios: 1.23 (4 nm); 1.06 (5 nm). These numbers are consistent with other literature reports [J. Cluster Sci., 12, 571–582 (2001); JACS, 135, 18536–18548 (2013)] and suggest a Cd-rich surface. A Cd-rich surface does not, however, necessarily suggest Se vacancies as the large Cd excess most likely arises from Z-type ligands on the NC surface. In fact, ascertaining the availability of sites arising from Se vacancies is definitely a challenge. We are postulating that we can infer the possibility via our XAFS measurements, but would never assert this is infallible.

** How is the charge balanced with substitutional and interstitial Cu atoms in the FEFF simulations? Is the interstitial Cu atom actually Cu(0)? Are there Cd vacancies?*

Author's reply: We thank the reviewer for their comment. The reviewer brings up a fantastic point…mainly how does one deal with charge in FEFF calculations. Charges, in general, are not handled well by FEFF. For the purposes of our calculations, we took a CdSe cluster and performed a number of operations to this cluster: we removed a Se or Cd atom, we added Cd and Cu interstitial

atoms, and we included a Cu atom substitutionally for Cd. In all cases, we did not remove others atoms (i.e. there were no Cd or Se vacancies). While we do understand that for every 2 interstitial Cu atoms we would require 1 Cd vacancy, we chose to exclude the inclusion of Cd vacancies.

The Cu atom is introduced formally as a Cu(0) atom, with a d^9 electron configuration. Now, during the SCF iterations during the FEFF calculations, FEFF will determine some amount of formal charge transfer between the angular momentum resolved states. In the case for interstitial or substitutional Cu, we observe ~0.7 electrons transferred to the Cu d-state, with a subsequent reduction in s-state count, suggestive of Cu(I)-type behavior, as expected for Cu dopants in CdSe.

Other than this, we do not introduce any type of formal charge neutralization during the calculation. While the reviewer does point to some areas of improvement, we note that the striking qualitative correlations between our simulations and experiment lends credence to our approach.

** How much of the Cd K-edge linewidth comes from inhomogeneous broadening from the ensemble?*

Author's reply: We thank the reviewer for their comment. We believe that the Cd K-edge linewidth can be primarily ascribed to instrumental broadening. We assert this for a few reasons:

1) The nominal resolution at 10-ID at APS is $\Delta E/E \sim 10^{-5}$. At 26.7 keV, this corresponds to $\Delta E \sim 0.3$ eV. This is actually an extremely impressive energy resolution at this energy range.

2) While we have an impressive energy resolution, we expect to observe no shifts in the Cd K-edge as a function of particle size. The Cd K-edge is probing transitions from 1s electrons into p-states 4-8 eV above the CBM. These are fairly flat bands that should be unaffected by quantum confinement [see band diagram below from our paper PRL, 98, 146803 (2007)].

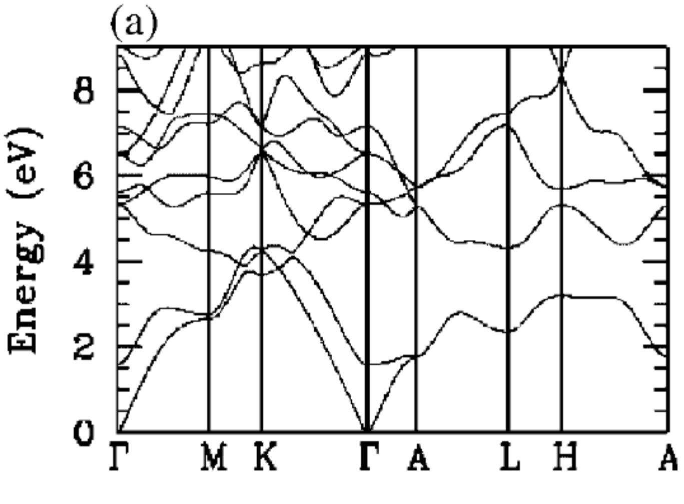

3) Even assuming some sort of shift in the Cd K-edge data, we would not expect the shift to be much greater than the nominal 0.3 eV resolution among the limited size ranges we studied in this manuscript.

** In the 'fully exchanged' Cu2Se Se K-edge XAFS spectrum shown in Figure 3b appears equivalent to the spectrum shown in Figure S10b with 950 Cu+ ions per NC. The corresponding Cd K-edge XAFS spectrum for this sample is shown in Figure S9b and does not appear to indicate CdSe nanocrystals are present, however, it is worthwhile trying to analyze the speciation of this presumably molecular Cd2+ byproduct. Have the authors tried to elucidate the identity of this Cd2+ species?*

Author's reply: We thank the reviewer for their comment. We were not able to elucidate the unique identity of this material. Our EXAFS analysis, however, suggests a 5 or 6-fold coordinate Cd species with a bond length of ~2.3 Å. Due to the by-products in the reaction vessel, likely reaction products could be cadmium hydroxide or some form of a cadmium oleate complex [J. Mater. Chem. C, 2, 5593 (2014)]. We expect that the formation of CdO is less likely. This was already discussed in the original manuscript.

Minor:

Please add the average size of the starting CdSe nanocrystals in Figure S13.

Author's reply: We thank the reviewer for their comment and apologize the oversight. The relevant size info has been added in the text box of Figure S13.

Reviewer #2 (Remarks to the Author):

The manuscript presented by Khammang, Wright and Meulenberg reports a study of Cd2+-to Cu+ cation exchange in CdSe nanocrystals by means of X-ray absorption spectroscopy techniques. Based on the results of the experiments the authors conclude that the reaction goes through interstitial inclusions of the guest cations in the host crystal lattice, one of the two debated mechanisms proposed to explain cation exchange in nanomaterials. The authors properly support and convincible proof their conclusions by carefully analyzing the experimental data. Nevertheless, in my opinion, this work does not have a potential to attract a broad interest of a research community reading the journal. First of all, in its current state the work is too specialized being focused solely on the analysis of (E)XAFS/XANES data. Second, the authors study only one certain system (i.e. Cu+-to-Cd2+ exchange), thus the results cannot be considered as general and applicable to other guest-host cations couples, especially, when nanocrystals possess a significant number of vacancies through which the diffusion of the both species can occur. Thus, this system is basically not representative. In addition, it has a questionable future in

terms of practical application, because 1) the use of CdSe NCs is significantly restricted (especially in Europe) and 2) such conversion from CdSe to Cu2Se does not make much sense since to date many synthetic protocols are developed directly providing Cu2-xSe NCs of excellent quality without the need for an additional cation exchange step. Third, as the authors correctly admit, cation exchange reactions depend on many parameters. Hence, even the same guest-host cations couple might behave differently depending on certain conditions, the nature of precursors, solvents, ligands, temperature, etc. To properly elucidate the mechanism of the exchange, several parameters should be considered and varied.

Author's reply: We thank the reviewer for their positive comment on our work. We see the reviewer does not think our results are generalized enough for publication in Nature Communications. First, we will note, and I am sure the reviewer will appreciate, the nature at which synchrotron radiation based research is undertaken. We only get a couple of beamtimes allocated per year and much planning must be done prior to the beamtime. In the case of the experiments in this paper, this took over one year of beamtime (3 different beamtimes). Our first beamtime yielded mainly zero positive results and was largely a "calibration" beamtime for us (i.e. testing the in situ cell, seeing what the nominal signal levels would be achieved with transmission vs fluorescence detection, etc.).

We learned much after the first beamtime and were able to get the data presented in the paper over the next 2 beamtimes. Near the end of the third beamtime, we felt satisfied with the copper cation exchange results and began examining Ag cation exchange in CdSe to begin to look at generalizability, at least in the same system. We were able to get some promising results, although we could not retrieve a full data set. Below, we plot some representative data for the Cd K-edge and Ag K-edge XANES and EXAFS for Ag cation exchange in 5.0 nm CdSe.

The Cd K-edge XANES data shows the same trends with increasing Ag concentration as observed with increasing copper concentration: an increase in the XANES "white line" along with isosbestic points. The Cd K–edge EXAFS data also shows the qualitative trends we discussed in our paper: a slight reduction and broadening of the nearest neighbor scattering contribution. At moderate Ag concentrations, we observe increased broadening, and at the highest Ag concentrations (i.e. conversation to Ag_2Se), we see a peak at low *R* which is suggestive of the Cd complex that is formed upon complete exchange.

Cd K-edge EXAFS

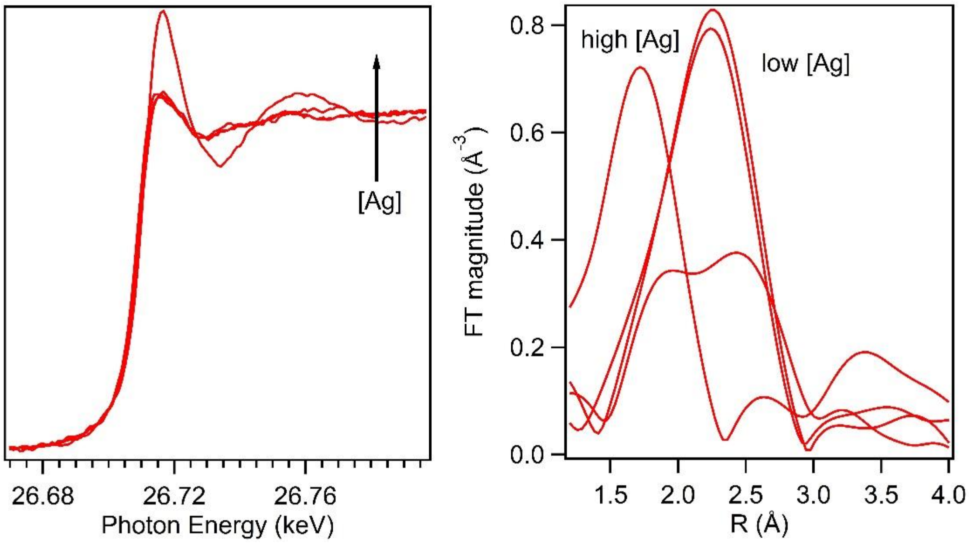

The Ag K-edge XANES data shows a similar trend to what we observed for the Cu K-edge XANES data. We see similar spectra features throughout the Ag concentration ranges, and the data look qualitatively similar to Ag_2S (we did not have a Ag_2Se standard, and have not yet been able to return to the synchrotron). In a more exciting manner, we were able to get decent quality EXAFS data for the Sg K-edge (which we unable to do with the Cu K-edge). The data is interesting as it shows a scattering component at ~2.8 Å (phase shift correct) at low Ag concentration for high Ag concentrations, we observe a prominent scattering component at ~2.1 Å. The EXAFS is qualitatively similar to the EXFAS for Ag_2S, but we have not performed any fits on the data at this time due to the incomplete nature of the data, and the fact the all relevant controls have not been able to be performed (for instance, the scattering feature at ~2.1 Å indeed related to Ag_2Se…or perhaps related to some other spurious Ag species that competes with Ag_2Se formation as colloidal stability of the converted species decreases which means we have more difficulties getting good signal at larger concentrations). We hope these preliminary results, however, show some form of generalizability to other dopant systems, and if the reviewer and/or editor request, we would be happy to include this data in the SI (with the disclaimer that it is not a fully completed set).

Ag K-edge EXAFS

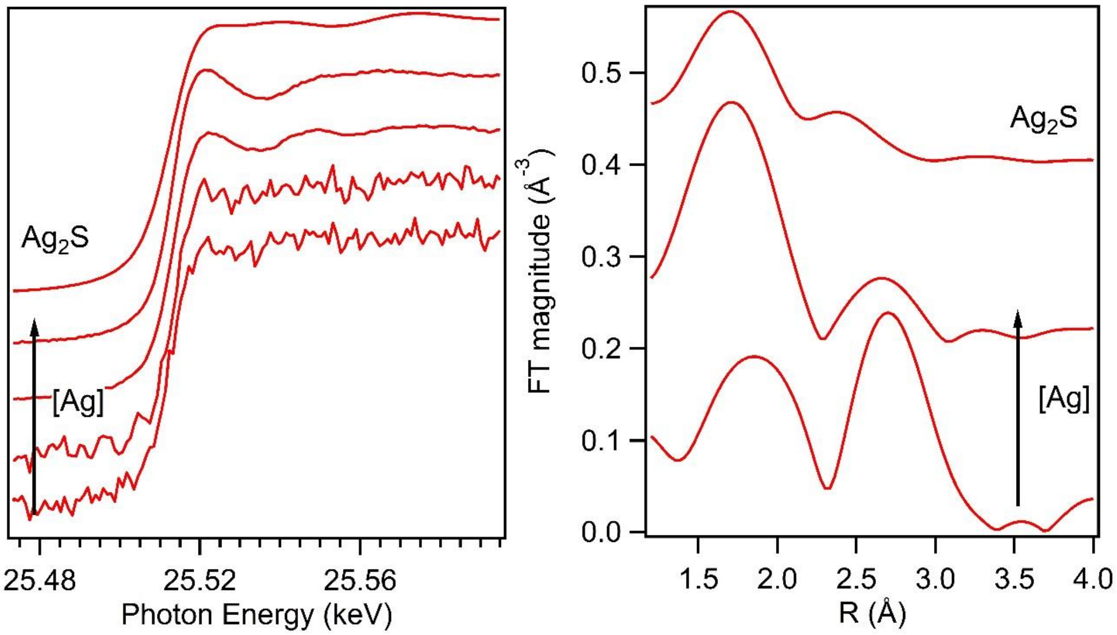

With regards to the cation exchange reaction depending on many parameters, we fully agree and stated this in the text, but we now realize we could have done a better job of this. For instance, we did try to change the solvent and cationic salts used to slow the reaction kinetics, but were unsuccessful. There is a vast parameter space to attack, and ideally it would be nice to be able to address them all.

It would be exciting to be able to follow up of these results with this parameter space in mind, but we are unable to return to the beamline due to the COVID pandemic. We hope, therefore, that our preliminary work on the Ag cation exchange in CdSe satisfy the reviewer in terms of showing some form of generalizability of our observations. I know demonstration on another semiconductor system would have been more preferred, but given our current limitations, I hope this is satisfactory.

The last point we will address is the specific system we chose to study. While we agree with the reviewer regarding the environmental aspect of CdSe, even the reviewer cannot deny CdSe is the archetypal NC system and therefore is the perfect material to study when trying to tease out subtle information.

Apart from these major issues, there are some minor points which lower the quality of the work. Among them are the following.

1. The language of the manuscript requires additional proof-reading and polishing. Buttery butter constructions like "The first report of CE in NCs reported…", "Initially suggested by the Alivisatos group[6], this model suggests …", "…these observations strongly suggest the observation …", etc. do not make the text more attractive.

Author's reply: We thank the reviewer for their comments. We apologize for the repetitiveness in the manuscript. We have edited parts of the text accordingly.

2. In the introduction the authors state that "The driving force (energetically) behind CE processes can be explained by considering hard/soft acid/base (HSAB) theory." This, in fact, only one of many parameters (summarized e.g. in Chem. Soc. Rev. 2013, 42, 89-96 and Chem. Rev. 2016, 116, 10852-10887), both thermodynamic and kinetic, affecting cation exchange. It is not entirely correct attributing the driving force to only one factor.

Author's reply: We thank the reviewer for their comments. We do agree that we there are multiple factors involved in the cation exchange process. In the case of our measurements, however, we are taking the kinetics out of the process by use a reactant limited reaction and allowing the reaction to equilibrate. The text has been revised accordingly.

3. The formula used by the authors to calculate concentration of CdSe NCs based on optical properties (from Chem. Mater. 2003, 15, 2854-2860) was reexamined and corrected later by the group of Mulvaney (J. Phys. Chem. C 2009, 113, 19468–19474). It is recommended to use this latest version for the corresponding calculations, which in turn may quantitatively change conclusions in this work.

Author's reply: We thank the reviewer for their comments. The reviewer picked up an error on our part. Concentration calculations were not performed via optical spectroscopy. Calculations were performed physically by weighing out an amount of nanocrystal and making a stock solution of the material (i.e. taking 50 mg of material and dispersing in 5 mL of solvent to make a 10 mg/mL stock solution). From this stock solution, dilutions were made, molar concentrations calculated, and the reactions were allowed to proceed. Since we were not using the extinction coefficients, and only the size correlation between exciton absorption (which is the same for both the Peng and Mulvaney paper), we do have any errors to correct, although we have added the Mulvaney reference for completeness. The text has been revised accordingly.

4. An image illustrating the mechanism of the exchange would be very useful to understand the process.

Author's reply: We thank the reviewer for their comments. We have included a figure that shows the CE process. If the editor feels this is not necessary, we would be happy to remove it.

Reviewer #1 (Remarks to the Author):

The authors have improved the manuscript and addressed all questions adequately for me to recommend this for publication.

Reviewer #2 (Remarks to the Author):

Even though the authors partly addressed my concerns about general applicability of their results initially obtained on Cd-to-Cu cation exchange reaction by exploring Cd-to-Ag exchange, the work in its current form remains too specific and lacks catchy points for a broad readership of the journal. In my opinion, it should be published in a more specialized journal.
